# Cytosine methylation changes in enhancer regions of core pro-fibrotic genes characterize kidney fibrosis development

**DOI:** 10.1186/gb-2013-14-10-r108

**Published:** 2013-10-07

**Authors:** Yi-An Ko, Davoud Mohtat, Masako Suzuki, Ae Seo Deok Park, Maria Concepcion Izquierdo, Sang Youb Han, Hyun Mi Kang, Han Si, Thomas Hostetter, James M Pullman, Melissa Fazzari, Amit Verma, Deyou Zheng, John M Greally, Katalin Susztak

**Affiliations:** 1 Renal Electrolyte and Hypertension Division, Perelman School of Medicine, University of Pennsylvania United States,, 415 Curie Blvd, Philadelphia, PA 19104, USA; 2 Department of Genetics, Albert Einstein College of Medicine, Bronx, NY 10461, USA; 3 Department of Pathology, Montefiore Medical Center, Bronx, NY 10461, USA; 4 Department of Epidemiology and Population Health, Albert Einstein College of Medicine, Bronx, NY 10461, USA; 5 Department of Oncology, Albert Einstein College of Medicine, Bronx, NY 10461, USA; 6 Case Western Reserve University, Cleveland, Ohio 44106, USA

## Abstract

**Background:**

One in eleven people is affected by chronic kidney disease, a condition characterized by kidney fibrosis and progressive loss of kidney function. Epidemiological studies indicate that adverse intrauterine and postnatal environments have a long-lasting role in chronic kidney disease development. Epigenetic information represents a plausible carrier for mediating this programming effect. Here we demonstrate that genome-wide cytosine methylation patterns of healthy and chronic kidney disease tubule samples obtained from patients show significant differences.

**Results:**

We identify differentially methylated regions and validate these in a large replication dataset. The differentially methylated regions are rarely observed on promoters, but mostly overlap with putative enhancer regions, and they are enriched in consensus binding sequences for important renal transcription factors. This indicates their importance in gene expression regulation. A core set of genes that are known to be related to kidney fibrosis, including genes encoding collagens, show cytosine methylation changes correlating with downstream transcript levels.

**Conclusions:**

Our report raises the possibility that epigenetic dysregulation plays a role in chronic kidney disease development via influencing core pro-fibrotic pathways and can aid the development of novel biomarkers and future therapeutics.

## Introduction

Clinical retrospective data indicate that altered nutrient availability during development could have a long lasting effect on the development of adult diseases, a phenomenon called 'programming'. Hypertension and chronic kidney disease (CKD) show one of the highest sensitivities to intrauterine programming [1]. Epigenetic changes caused by altered intrauterine nutrient availability have been proposed as the mechanistic link for hypertension and CKD development [2]. Epigenetic modifications are inherited during cell division, thus solidifying 'the memory or programming' effects of the environment [3]. The epigenome, which includes the covalent modifications of DNA and its associated proteins and defines DNA accessibility to the transcriptional machinery, is the key determinant of outcome after transcription factor binding. At the root of the epigenetic modifications is the direct chemical modification of cytosines by methylation [4]. In different cancer types, hypermethylation of tumor suppressor gene promoters has been observed [5]. Increased promoter methylation can interfere with transcription factor binding, causing loss of tumor suppressor expression, thereby contributing to the malignant transformation [6,7]. Agents that reduce cytosine methylation (for example, azacytidine) are now in clinical use and are associated with improvements in clinical outcome, especially for patients with myelodysplastic syndrome [8]. In addition, mutations of different chromatin-modifying enzymes have been described in various cancer types, contributing to alterations in the cancer epigenome [9].

## Background

Not much is known about the epigenome of chronic human diseases other than cancer. Most previous studies have been performed on cultured cells, animal models, or surrogate cell types (mostly circulating mononuclear cells) [10]. As the epigenome is cell type-specific, little mechanistic information can be drawn from cultured cells and surrogate cell types [11]. To understand whether or not epigenetic changes occur and thereby potentially contribute to CKD development in patients, we performed genome-wide cytosine methylation profiling of tubule epithelial cells obtained from CKD and control kidneys. We found that core fibrosis-related genes show cytosine methylation changes in their gene regulatory regions. *In vitro* studies indicate that cytosine methylation differences play a role in regulating transcript expression. Examining the CKD epigenome can be an important first step in understanding the role of epigenetics outside the cancer field [12].

## Results

### CKD kidneys show distinct cytosine methylation profiles

Human kidney samples were collected from healthy living transplant and surgical nephrectomies and categorized based on their clinical and pathological characteristics (Table 1; Additional file 1). In the initial dataset we combined hypertensive and diabetic CKD as cases, since the clinical, histological and gene expression profiles of these samples were highly similar (Additional file 2). In the replication dataset, only diabetic CKD (DKD) samples were used. In both datasets, the criteria for controls were an estimated glomerular filtration rate (eGFR) greater than 60 cc/minute/1.73 m^2^, absence of significant proteinuria, and less than 10% fibrosis on histology. Samples with significant hematuria or other signs of glomerulonephritis (HIV, hepatitis or lupus) were excluded from the analysis. In summary, 26 samples were used for the initial discovery phase and the phenotype analysis was significant for racial diversity and included subjects with and without diabetes both as cases and controls (Table 1; Additional file 1).

**Table 1:** Demographic, clinical and histological characteristics of the samples

**Characteristics**	**Diseased**	**Healthy**	***P*-value**
**n**	**12**	**14**	
Age (years) mean ± SD	68.0 ± 10.81	61.14 ± 11.2	0.11
Ethnicity			
Asian, Pacific Islander	0	1	
White, non-Hispanic	4	2	
Black, non-Hispanic	4	4	
Hispanic	1	3	
Other and unknown	3	4	
Height (cm) mean ± SD	165 ± 8.69	166.5 ± 8.63	0.6
Weight (kg) mean ± SD	78.0 ± 22.02	88.32 ±15.93	0.2
BMI (kg/m^2^) mean ± SD	27.85 ± 6.41	31.25 ± 5.58	0.18
Diabetes	6	5	
Hypertension	11	12	
Proteinuria (dipstick)	3.0 ± 1.83	0.36 ± 0.81	1.80E-04
Serum BUN (mg/dL) mean ± SD	35.0 ± 14.7	17.71 ±5.85	4.60E-04
Serum creatinine (mg/dL) mean ± SD	3.0 ± 1.61	1.08 ± 0.18	2.00E-03
eGFR (ml/minute/1.73 m^2^) mean ± SD	29.0 ± 13.68	70.94 ± 8.35	1.06E-09
Histology			
Glomerulosclerosis (%)	31.0 ± 31.35	3.31 ±5.52	4.00E-03
Mesangial matrix expansion	1 ± 0.91	0.17 ± 0.39	0.03
Tubular atrophy (%)	34.0 ± 24.94	9.82 ± 15.76	6.00E-03
Interstitial fibrosis (%)	34.0 ± 25.15	5.68 ± 5.07	4.00E-04
Vascular sclerosis			
Intima	2.0 ± 0.78	0.9 ± 1.1	1.50E-03
Arterioles	2.0 ± 0.78	0.29 ± 0.62	4.00E-04

eGFR, estimated glomerular filtration rate; SD, standard deviation.

**Figure 1:**
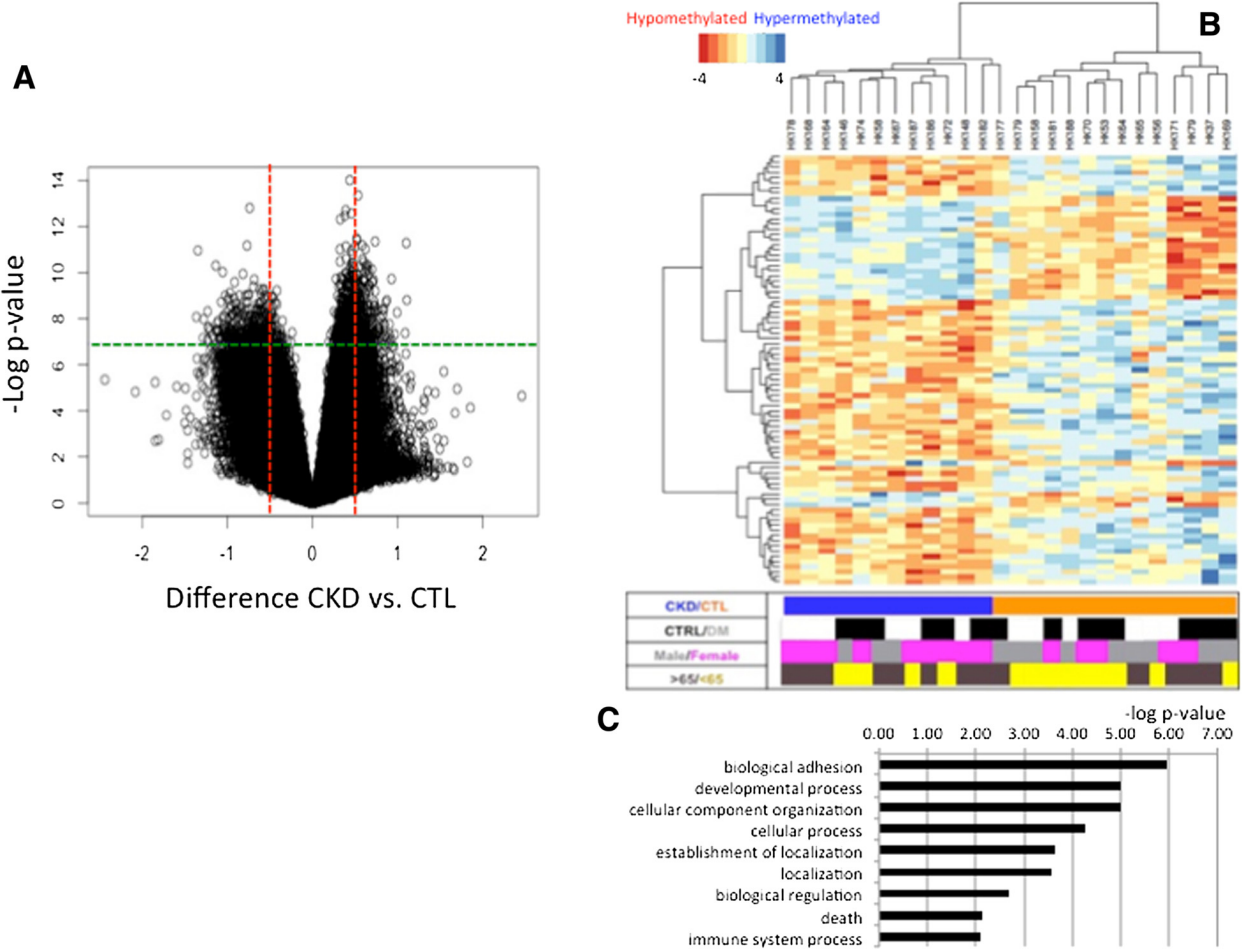
**Statistically significant cytosine methylation differences in chronic kidney disease. (A)** Volcano plot analysis of cytosine methylation differences. The x-axis represents the relative cytosine methylation difference of control (CTL) versus CKD samples, the y-axis represents the negative log_2_ of the *P*-value of that locus. The mean *P*-value and mean difference of 1.3 million loci present on the chips are plotted on the graph. The green and red lines represent the statistical criteria used for further analysis (*P*-value and fold change, respectively). **(B)** Hierarchical cluster analysis of the differentially methylated regions. Each column represents changes from one individual kidney sample; blue indicates hypermethylation in CKD, while red represents hypomethylation in CKD. The chart below shows the clinical parameters of the samples: glomerular filtration rate, diabetes status (DM, diabetes mellitus), sex, and age (aged >65 years or <65 years). **(C)** Gene Ontology analysis of the 1,535 DMRs mapped to unique genes using DAVID gene ontology annotation groups (biological process level 1 annotation).

To avoid cell-type heterogeneity, we microdissected each renal cortical sample and used the tubular epithelial cell portion for the initial analysis [13]. Our and other labs previously published that this fraction represents mainly the proximal tubule portion of the human kidney [13]. Genome-wide cytosine methylation analysis was performed on each sample using methylation-sensitive and -insensitive isoschizomer enzymes (HpaII and MspI) followed by (HpaII) fragment enrichment by ligation-mediated PCR (HELP) [14]. Samples were hybridized on Nimblegen whole genome-covering microarrays (1.3 million loci). Focusing on loci that showed more than 50% difference in their methylation ratio and a *P*-value <0.01, we identified 4,751 differentially methylated regions (DMRs) between control and diseased tubule samples (Figure 1A; complete list provided in Additional file 3). The volcano plot analysis (fold change of methylation plotted against the negative log_2_ of the *P*-value) indicated that 70% of the DMRs showed lower methylation level in CKD (Figure 1A). We found that cytosine methylation differences suffice for proper clustering and supervised classification of control and CKD kidney samples (Figure 1B). The computational annotation identified a total of 1,535 unique genes in the vicinity of the DMRs.

Gene ontology annotation showed that genes around the DMRs are enriched for cell adhesion and development related functions including: collagen, fibronectin, transforming growth factor beta (TGFβ) and Smad proteins (Figure 1C), many of these genes are known to play a critical role in CKD development. In summary, microdissected kidney tubule cells showed distinct differences in their cytosine methylation patterns in CKD.

### Validation and external replication of the results

Internal validation of the results was performed using site-specific primer-based amplification of bisulfite-converted genomic DNA and MassArray Epityper (Sequenom) quantification of modified cytosines [15]. This method is based on mass spectrometry that allows us to determine absolute methylation levels. We correlated these (mass array based) absolute methylation levels with the HpaII/MspI relative ratios (Additional file 4).

**Figure 2:**
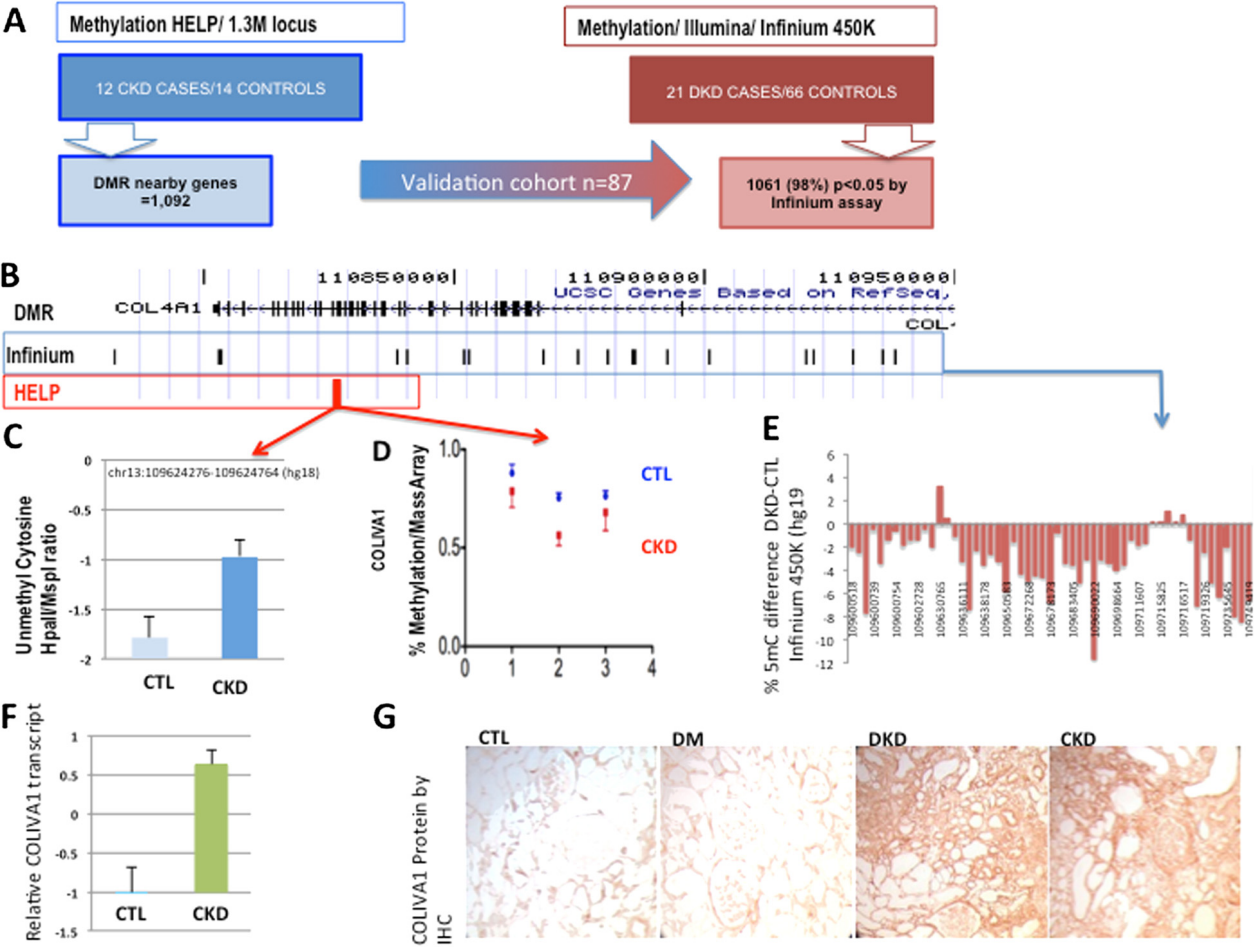
**External and internal validation of the observed changes. (A)** Correlation of the DMRs identified in 26 samples using the HELP method and an external dataset containing 87 human kidney samples analyzed using Illumina Infinium 450K arrays. We found concordant regulation of 1,061 transcripts (98%) from the 1,092 mapped genes using this validation dataset. Of transcripts that showed both differential methylation and expression, 404 (97%) were confirmed. **(B)** An example of a DMR identified by the HELP assay and confirmed in the validation dataset. This DMR is localized in the intronic region of *COLIVA1*. **(C)** Methylation status and **(D)** gene expression of *COLIVA1* in the original HELP dataset in control and CKD samples. **(E)** MassArray confirmation of methylation status of the *COLIVA1* locus (blue is mean ± standard deviation of control samples, red is mean ± standard deviation of CKD samples). **(F)** Methylation status of the *COLIVA1* locus in the validation dataset (Infinium 450K arrays). The data represent the mean differences in absolute methylation levels of individual cytosines at the *COLIVA1* locus. **(G)** Immunohistochemistry of *COLIVA1* expression in control (CTL), diabetic (DM), DKD and CKD kidneys.

External validation was performed on 87 microdissected human kidney tubule epithelial samples, 21 samples from patients with DKD and 66 controls (including hypertension (n = 22), diabetes mellitus (n = 22) or none (n = 22)) (SYH and KS, unpublished observation). Genome-wide methylation profiling of the validation set was performed using Illumina Infinium 450K methylation-sensitive bead arrays. This method uses site-specific probes for bisulfite-converted DNA, which is fundamentally different from the restriction enzyme-based analysis used in the HELP analysis. From the 1,535 unique genes found around DMRs in the initial dataset, we examined 1,092, as these genes were present also on the Illumina Infinium (and Affymetrix expression) arrays (Figure 2A).

Significant methylation differences were detected for 1,061 genes (corresponding to 98% of the genes in the original dataset; Figure 2A). The complete list of DMRs in the original and the replication dataset can be found in Additional file 5.

Locus-specific validation was performed for six different genes, including *COLIVA1*. COLIV4A1/A2 are critical basement membrane proteins synthesized by epithelial cells. Increased expression is known to be responsible for increasing the thickness of the basement membrane and it is considered to be an early change in progressive kidney fibrosis [16]. The *COLIVA1* and *COLIVA2* transcripts are transcribed from a single promoter (Figure 2B). This locus showed significantly lower cytosine methylation of CKD samples (Figure 2C). We examined the absolute methylation level of *COLIV4A1/2* by MassArray Epityper analysis (Figure 2D) in control and CKD samples and confirmed the methylation differences between healthy and diseased tubule epithelial cells. Next we examined *COLIVA1/2* methylation in the validation dataset (Infinium arrays from 66 control and 21 DKD samples). Using this dataset we also confirmed the predominant (2 to 12%) hypomethylation of this locus (Figure 2E). The methylation differences correlated with increased *COLIVA1* transcript (Figure 2F) and protein levels (Figure 2G). Using the MassArray Epityper we also validated the methylation status of additional loci (Figure S3A,B in Additional file 6). In summary, the methylation differences appear to be highly consistent between the original and validation experiments using multiple different methods.

### Differentially methylated loci are enriched in kidney-specific gene regulatory regions

**Figure 3:**
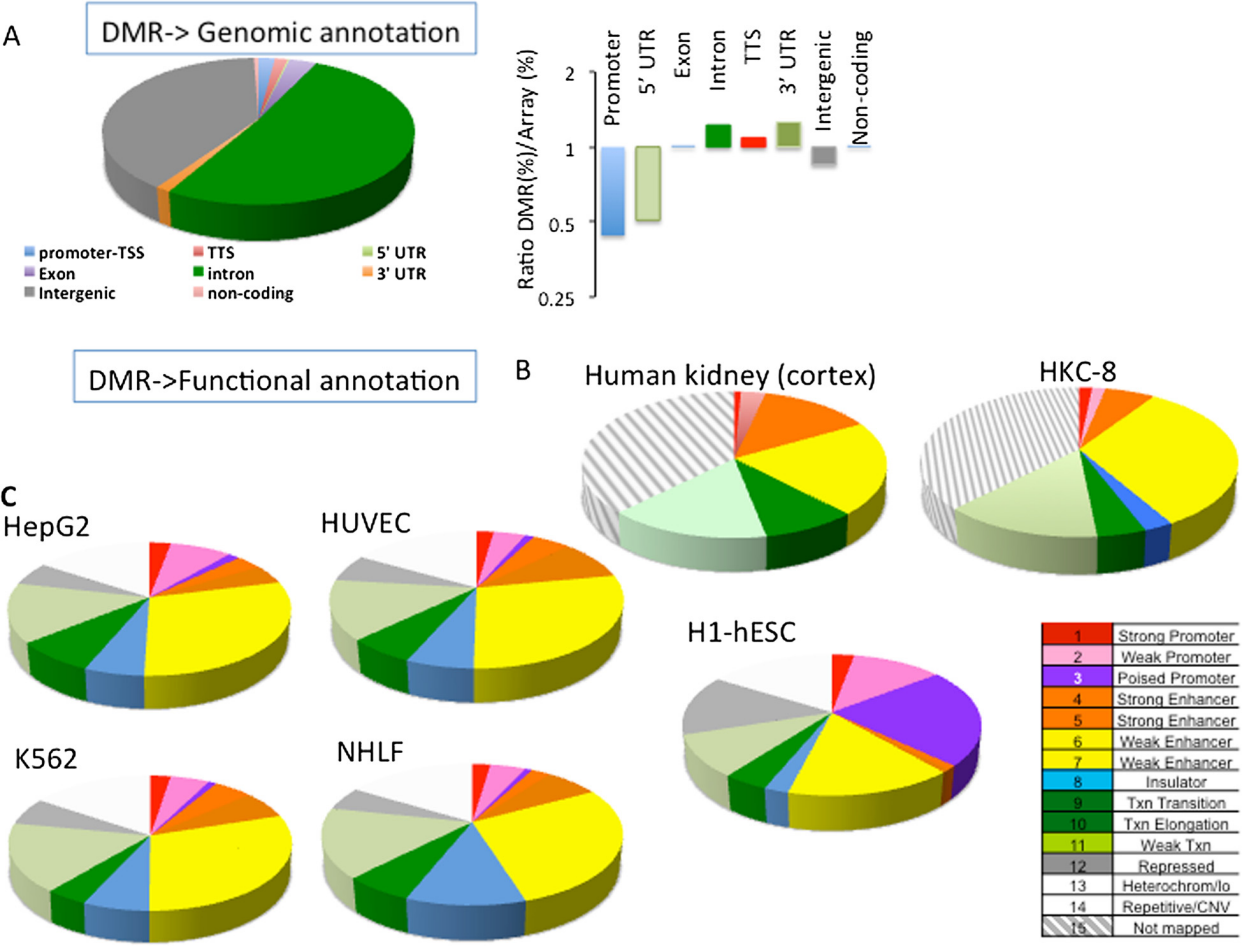
**Chronic kidney disease differentially methylated regions are localized to kidney-specific enhancer regions. (A)** RefSeq annotation of the DMRs. Relative enrichment ratio of the DMRs compared with the representation of the different elements on the methylation microarray. TSS, transcription start site; TTS, transcription termination site. **(B)** DMRs overlap with regulatory element (chromatin state) annotation maps of renal tubule epithelial cells (HKC8) and adult kidney cortex, indicating that most differentially methylated cytosines are localized to enhancer (yellow and orange) regions in kidney epithelial cells. The color code annotation of the chromatin state map is provided bottom right. **(C)** Chromatin annotation of the DMRs in five different ENCODE cell lines (H1, human embryonic stem cells; HepG2, hepatocytes; HUVEC, endothelial cells; K562, erythroid cells; NHLF, human lung fibroblasts).

Cytosine methylation of promoters is critically important as it can interfere with transcription factor binding and thereby modulate transcription [7]. The number of DMRs localized to RefSeq annotated promoters and 5′ UTRs was significantly (about 50%) lower than the expected ratio (Figure 3A). On the other hand, more than half of the DMRs were in gene body-related regions. Only a few DMRs localized to exons (approximately 200); the majority of the differences we observed are in the intronic regions (Figure 3A). We also examined the RefSeq annotated genomic distribution of the hypo- or hypermethylated regions (Additional file 7). The percentage of hypermethylated regions was similar in the different RefSeq-based annotation groups. We found that more loci showed increased methylation at the 3′ UTR (Additional file 7). In summary, the genomic regions that showed differences in their cytosine methylation pattern in CKD were not promoters, but intronic and transcription termination regions and 3′ UTRs.

To further understand the functional significance of the DMRs, we generated genome-wide chromatin annotation maps using cultured human proximal tubular epithelial cells (HKC8). First, we performed chromatin immunoprecipitation followed by next-generation sequencing (ChIP-seq) for a panel of important histone modifications: H3K4me1, H3K4me2, H3K4me3, H3K27ac, H3K27me3, and H3K36me3. Next, we generated gene regulatory annotation maps from the panel of ChIP-seq data using the hidden Markov model-based ChromHMM chromatin segmentation program [17,18]. Consistent with the RefSeq-based annotation, there are very few DMRs localized to ChromHMM-annotated kidney promoter regions (Figure 3B). The analysis indicated that 30% of the DMRs localized to enhancer regions, which was the most significant enrichment. Similar results were obtained when we generated adult kidney cortex ChromHMM maps (from published ChIP-seq data; Figure 3B) [19]. Next, we compared CKD-specific DMRs with chromatin annotation maps of other, different cell types using the publicly available ENCODE database (Figure 3C). We found that CKD-specific DMRs localized mostly to repressed chromatin regions, while transcription and enhancer regions showed the second highest enrichment. The result indicates that DMR in CKD are enriched in kidney-specific gene regulatory regions, mainly (intronic) enhancers.

**Figure 4:**
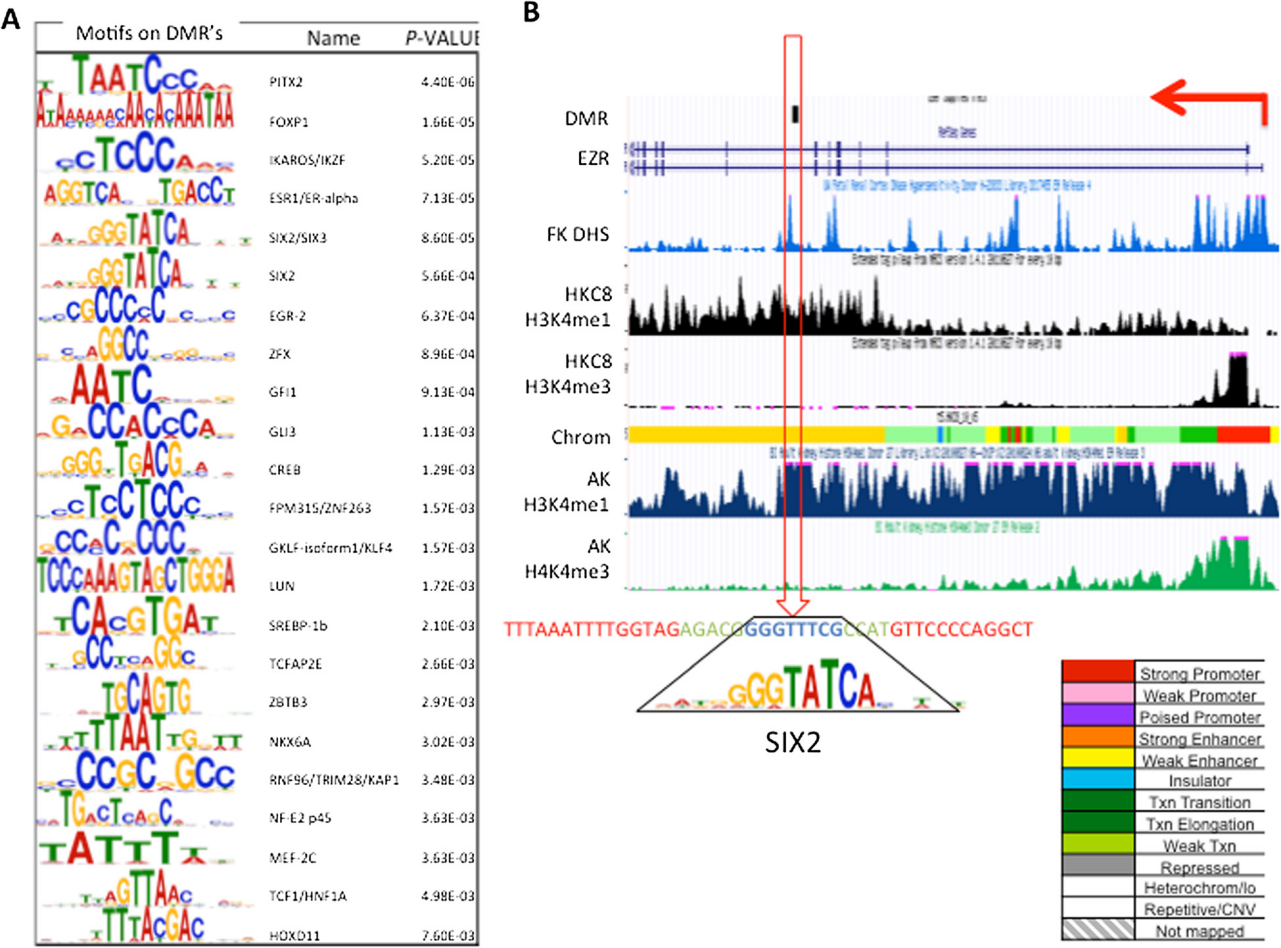
**Chronic kidney disease differentially methylated regions are enriched for kidney-specific transcription factor binding sites. (A)** The DMR and DHS sites contain consensus sequences. The transcription factor binding site motifs and their statistical enrichment from the *de novo* searched consensus sequences in DMR and DHS sites. **(B)** A specific example of an intronic DMR (within the *EZR* gene). The genomic location of the DMR is at the top, followed by the RefSeq representation of *EZR*; fetal kidney (FK)-specific DHS tracks (in blue); HKC8 cell-specific H3K4me1 and H3K4me3 tracks; HKC8 cell specific ChromHMM annotation of the locus (yellow, enhancer; red, promoter; green, transcription-associated region; the full color coding key is shown bottom right) - the sequences contain consensus-binding sites for the key kidney transcription factor SIX2/3, with the SIX2/3 binding motif illustrated as a sequence logo plot below; and adult kidney (AK)-specific H3K4me1 (blue) and H3K4me3 (green) tracks [19].

Gene regulatory regions are usually characterized by DNase I hypersensitivity (DHS) [20] as DNA is usually histone-free in gene regulatory regions so transcription factors can bind to these regions. Therefore, we overlapped the DMRs with human fetal kidney and human proximal tubule epithelial cell DHS-seq data (Gene Expression Omnibus (GEO) accession GSM530655). The statistical analysis confirmed that DMRs are enriched in DHS sites in both the fetal kidney epithelial dataset and the cultured tubule epithelial cell dataset (data not shown). Furthermore, we examined whether DMRs that overlap with DHS sites show similarities, by identifying the top 10 consensus sequences using the MEME software [21]. To search for transcription factor binding motifs amongst the top 10 sequences, we map the sequences to the JASPAR, UniProbe and Transfac databases [22]. The analysis highlighted that the DMRs contain consensus-binding sequences for transcription factors that play important roles in proximal tubule development, including SIX2, HNF, and TCFAP. The list of computationally identified transcription factor consensus motifs is shown in Figure 4A. Figure 4B illustrates our complex computational analysis. Here a DMR is located in the intronic region of the *EZR* (ezrin) gene. The DMR overlapped with adult kidney and renal tubular epithelial cell-specific H3K4me1 histone modification, but not with H3K4me3 enrichment. H3K4me1 is a specific histone tail modification for enhancer regions, while H3K4me3 is a marker of promoters. The ChromHMM-based gene regulatory region annotation confirmed that this region is an enhancer in renal epithelial cells (yellow region in the genome). These results indicate that by multiple different approaches this DMR is located in a gene regulatory region, an enhancer (Figure 4B). In addition, this region contained a consensus binding sequence for SIX2, further confirming that this is a gene regulatory region. In summary, our results indicate that CKD-specific DMRs are located in non-promoter gene regulatory regions, mainly enhancers, and contain consensus-binding motifs for renal-specific transcription factors.

### Differential methylated regions are functionally relevant and correlate with transcript levels

**Figure 5:**
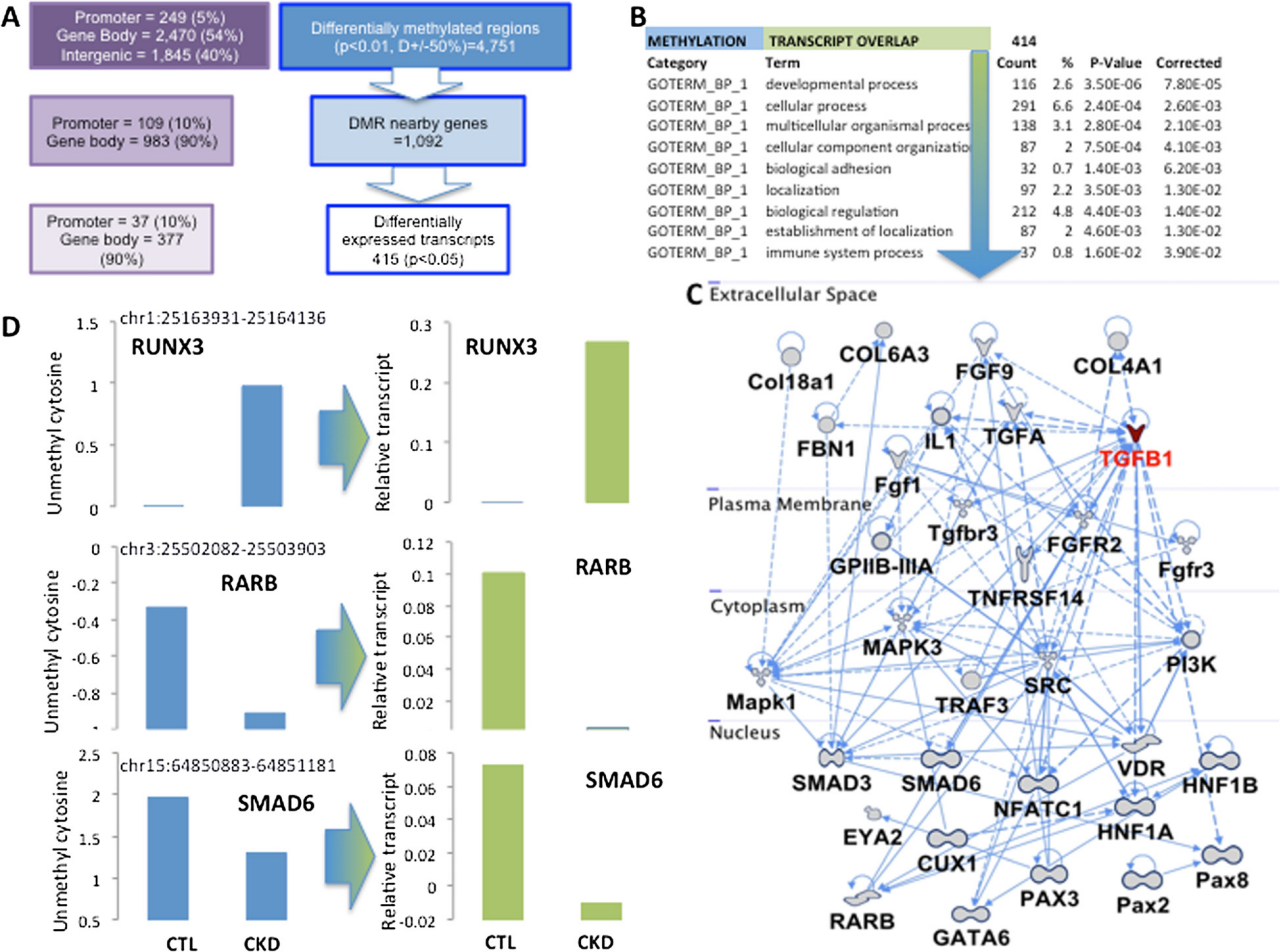
**Differentially methylated regions correlate with transcript changes. (A)** The 4,751 DMRs mapped to 1,092 unique genes that were present in the Affymetrix arrays. There were 415 transcripts that showed differences both in their methylation status and their expression in CKD samples. The RefSeq-based locations of the DMRs are also shown. While most differentially methylated regions localize to gene body regions, they also show correlation with the expression of many of those genes. Not only are the 415 transcripts differentially expressed, they also show differences in their cytosine methylation profiles as well. **(B)** DAVID-based gene ontology annotation of the 415 transcripts. **(C)** Network chart of the genes that are both differentially expressed and methylated). **(D)** Methylation and gene expression level of key molecules (RUNX3, RARB, SMAD6) identified by the network analysis in control (CTL) and CKD samples.

Next, we studied the functional significance of DMRs. First, we examined whether they correlate with downstream transcript levels. Gene expression changes were analyzed using RNA samples extracted from the same microdissected tubule samples used in the methylation assay. Individual RNA samples were hybridized to Affymetrix U133 arrays and the data ware normalized and analyzed using established pipelines [13]. From the 1,092 transcripts that were in close proximity to the DMR regions (Figure 5A), we found 415 (approximately 40%) genes showing significantly differential expression in the CKD samples (Figure 5A). As most DMRs were in non-promoter regions, most transcript changes correlated with intronic DMRs (Additional file 8). Gene ontology and network analyses highlighted differences in cell adhesion (collagens and laminins) and development-related pathways (Figure 5B,C). Specifically, we observed significant enrichment for differential expression and methylation in the TGFβ pathway, especially in *TGFBR3*, *SMAD3*, *SMAD6* and other targets (Figure 5D). These genes are known to be critical in CKD development [23,24]. In summary, cytosine methylation changes showed correlation with gene expression differences and identified concordant changes in the TGFβ pathway, a well-known regulator of kidney fibrosis development.

### Differentially methylated region methylation drives gene expression *in vitro*

To further dissect the relationship between cytosine methylation and transcript level changes, we analyzed gene expression and cytosine methylation patterns of tubule epithelial cells at both baseline and 9 days after treatment with a DNA methyltransferase inhibitor, decitabine (5-aza-2-dexoycytidine). We used AffymetrixST1.0 arrays to compare gene expression changes and the Infinium 450K arrays to examine cytosine methylation changes in control (n = 3) and decitabine-treated cells (n = 4).

**Figure 6:**
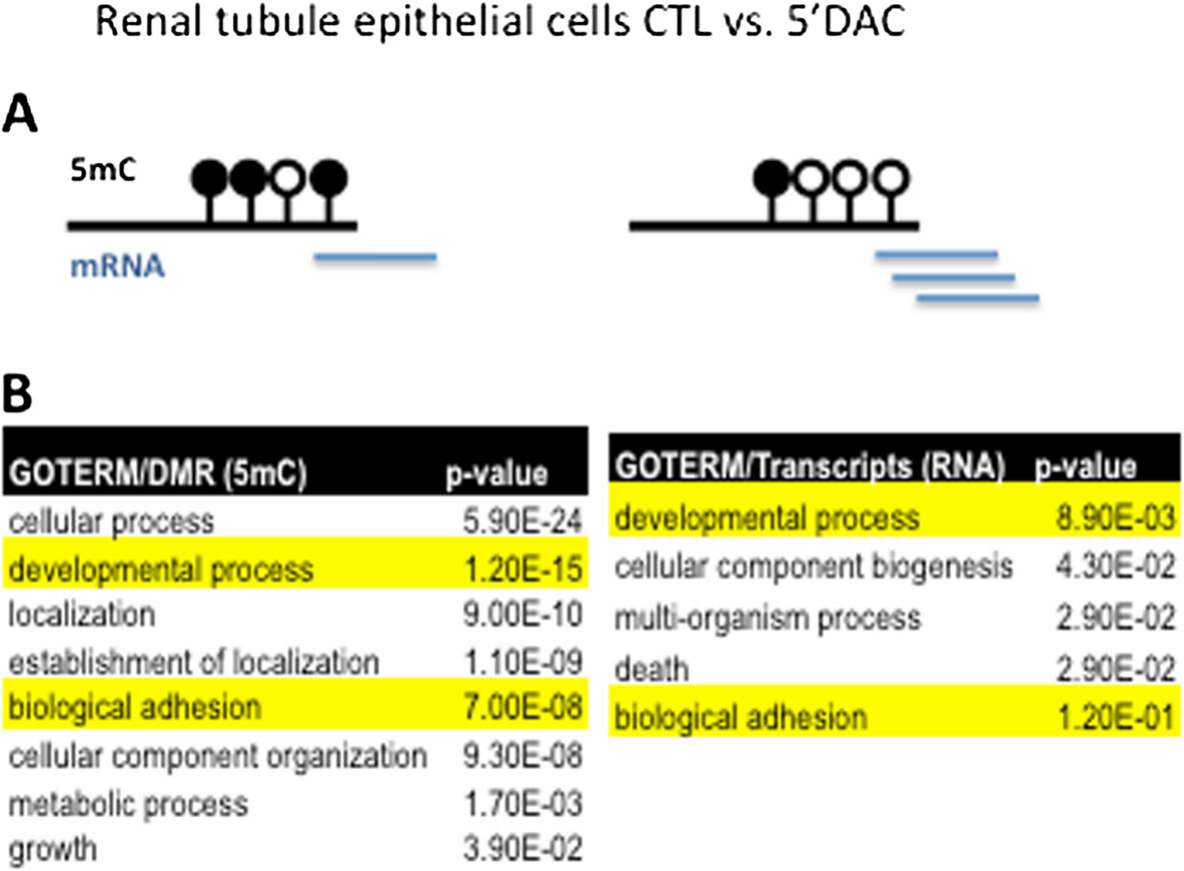
**Regulation of transcripts by a DNA methyltransferase inhibitor in *in vitro *cultured human tubular epithelial cells.** Gene ontology terms of transcripts showing differential expression in the decitabine-treated cells. **(A)** Illustration of regions that showed differential methylation of cultured HKC8 cells treated with 0.5 μM decitabine (5'DAC). CTL, control. **(B)** The interconnected network analysis highlighted the differential expression of cell adhesion and developmental pathways. These genes are also differentially expressed and methylated in the original CKD dataset. GO, gene ontology.

We tested whether we can identify a correlation between DMR and gene expression changes observed in CKD (*in vivo*) and gene expression and methylation changes *in vitro* after decitabine treatment. Decitabine is a cytosine analogue; therefore, we can safely assume that after decitabine treatment the cytosine methylation changes were the primary cause for transcript level changes. A limitation of the experiment is that decitabine induces demethylation of genomic loci that could be different from the CKD DMR. Large numbers of loci showed concordant differential methylation and gene expression changes in CKD (*in vivo*) and following (0.5 μM) decitabine treatment (*in vitro*), indicating that cytosine methylation changes in CKD might be the functional drivers for transcript level changes (Additional file 9). Genes related to cell adhesion (for example, collagen molecules) showed differential methylation following decitabine treatment as well (Figure 6A,B). In addition, just as we observed before, we found that genes related to development and cell adhesion were also differentially expressed following decitabine treatment (Figure 6B,C).

**Figure 7:**
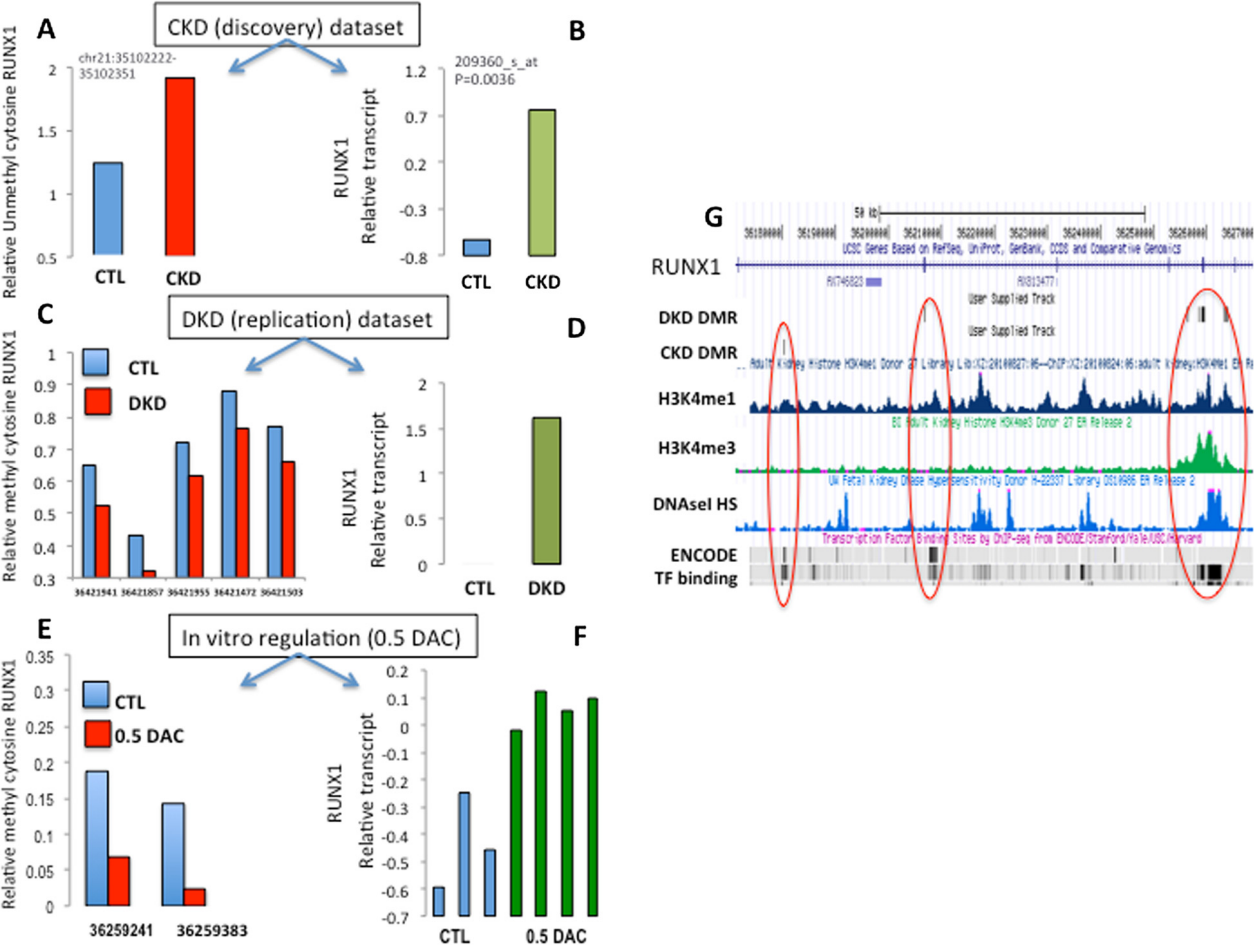
**Gene body cytosine methylation changes drive gene expression differences. ***RUNX1* methylation and gene expression were examined only **(A,B)** In the original discovery dataset, the gene body region of *RUNX1* was hypomethylated (A) and the corresponding transcript level was increased (B) in the CKD (discovery) dataset. CTL, control. **(C,D)** The differential methylation (C) and expression (D) of *RUNX1* in the DKD replication dataset. **(E,F)** Transcript levels are increased (F) *in vitro* in cultured tubules after decreasing the methylation level of the locus following 0.5 μM decitabine (DAC) treatment (E). **(G)** Genomic representation of the *RUNX1* locus showing DMRs in the DKD dataset and in the CKD dataset. Different tracks are shown for the *RUNX1* locus, including RefSeq gene, DMRs in the DKD dataset, DMRs in the CKD cells, and histone ChIP-seq data for H3K4me1 and H3K4me3 for adult kidney cortex and DHS sites from fetal kidneys. In addition, ENCODE-based transcription factor binding sites are also shown.

SMAD3 appears to be one of the most important mediators of the pro-fibrotic effect in the TGFβ and angiotensin II pathways [23]. The *SMAD3* locus contained DMRs in both the initial and validation datasets (data not shown). *SMAD3* expression levels were lower in both the original and confirmation datasets. Decitabine changed the methylation of this locus and subsequently it also changed *SMAD3* transcript levels (data not shown). To illustrate our findings, while *RUNX1* clearly plays an important role in leukemia development, it is expressed in both mouse and human in the developing and adult kidneys [25]. *RUNX1* was also shown to be differentially expressed in CKD tubules [13]. Both the original and replication dataset showed differential methylation of this locus (Figure 7A,C) and *RUNX1* transcript levels were increased in both datasets (Figure 7B,D). *RUNX1* DMRs clustered in ChromHMM annotated enhancer regions (Figure 7G) as it localized to H3K4me1 and DHS regions. *In vitro* treatment with decitabine changed the cytosine methylation of this locus, and the changes overlapped with the enhancer DMRs (Figure 7E). Subsequent to the DMR change of this locus, we also observed an increase in *RUNX1* transcript levels both *in vivo* and *in vitro* (Figure 7F). As decitabine did not change the methylation of the *RUNX1* promoter and affected only the methylation levels of the enhancer site, the result potentially indicates a causal relationship between enhancer-related DMRs and gene expression changes. The concordant changes in cytosine methylation and gene expression in CKD and *in vitro* (following DNA methyltransferase inhibitors) indicate that DMRs are potential drivers of critical CKD gene expression.

## Discussion and conclusion

While epigenetic dysregulation has been suggested as a mechanism for the development of many diseases, little is known about the epigenome of normal and diseased human cells and organs. Here we describe cytosine methylation differences in tubule cells obtained from patients with CKD. We found that CKD DMRs have many special features. First, most loci showed consistent cytosine methylation differences in different forms of CKD. These changes were smaller compared with what has described in the cancer literature previously. While the absolute differences were modest, the identified loci showed highly consistent changes even across different datasets and platforms. Unexpectedly, we found that most methylation differences localized outside of promoter areas, with promoter regions markedly spared from cytosine methylation differences. Our results indicate that the differentially methylated regions were located mainly at candidate enhancers. We found that the DMRs contain consensus-binding motifs for key renal transcription factors (HNF, TCFAP, SIX2). Furthermore, cytosine methylation levels correlated with baseline gene expression changes. These epigenetically distinct but morphologically similar cells also showed differences in their cytokine response. We illustrated our findings in a model hypothesizing that enhancer DMRs might modify transcription factor binding and thereby downstream transcript levels.

Based on our results, we propose that cytosine methylation changes are causally linked to transcript levels and phenotype development. As hypertensive and diabetic tubule samples showed similarities (both in cytosine methylation and gene expression changes), the observed changes are likely to be part of a common mechanism of progression. This may be expected, as phenotypically the tubulointerstitial fibrosis of DKD and hypertensive CKD is similar. In addition, we found that DMRs were enriched for genes related to development, many of them no longer expressed in the adult kidney. The DMR regions also contained binding sites for key kidney developmental factors (such as SIX2, HNF, and TCFAP). One possible interpretation of our findings is that the epigenetic differences are established during development. This is the time when the cell type-specific epigenome is established and when these genes and transcription factors play functional roles. Therefore they can possibly provide the mechanistic link between fetal programming and CKD development - the Brenner-Barker hypothesis put forward many decades ago [26,27], proposing that nutrient availability during development has a long lasting programming role in hypertension and CKD development. In addition, reactivation of the developmental pathways is also needed during organ injury repair [28]. We can also speculate that the altered developmental wiring of these pathways could continue to play a role later on as alterations observed after repair. Indeed, control and CKD kidney epithelial cells showed not only cytosine methylation differences but also different responses to cytokine treatment.

A limitation of our results remains that our samples were collected in a single center. Furthermore, base pair resolution results will likely help to refine the more precise location of DMRs and the methylation differences in the future. Furthermore, while microdissection is an excellent separation method to generate a homogenous tubular epithelial cell population from the kidney, the potential risk for increased cell type heterogeneity in CKD remains. As isolated and cultured cells continued to show many of the epigenetic and transcriptional differences, it is more likely that the observed differences are not related to cell type heterogeneity.

In summary, while it has long been speculated that epigenetic dysregulation might occur in non-cancerous diseases, including CKD, here we provide experimental evidence for cytosine methylation changes in human kidney tissue samples, opening the possibility that they play a role in CKD development.

## Materials and methods

### Ethics statement

The clinical study used the cross-sectional design. Kidney samples were obtained from routine surgical nephrectomies. Samples were de-identified and the corresponding clinical information was collected by an individual who was not involved in the research protocol. The study was approved by the Institutional Review Boards of the Albert Einstein College of Medicine Montefiore Medical Center (IRB#2002-202) and the University of Pennsylvania. Histological analysis was performed by an expert pathologist (IRB#815796).

### Tissue handling and microdissection

Tissue was placed into RNALater and manually microdissected at 4°C for glomerular and tubular compartments as described earlier. Dissected tissue was homogenized and RNA was prepared using RNAeasy mini columns (Qiagen, Valencia, CA, USA) according to the manufacturer’s instructions. RNA quality and quantity was determined using Lab-on-Chip Total RNA PicoKit (Agilent BioAnalyzer, Santa Clara, CA,USA). Only samples without evidence of degradation were used. Genomic DNA was extracted by phenol chloroform protocol for HELP analysis and the DNAeasy kit was used for the Infinium platform.

### DNA methylation analysis by HELP

The HELP assay was carried out as previously published [29]. Intact DNA of high molecular weight was corroborated by electrophoresis on 1% agarose gels in all cases. One microgram of genomic DNA was digested overnight with either HpaII or MspI (NEB, Ipswich, MA, USA). The digested DNA was used to set up an overnight ligation of the HpaII adapter using T4 DNA ligase. The adapter-ligated DNA was used to carry out the PCR amplification of the HpaII- and MspI-digested DNA as previously described [14]. Both amplified fractions were submitted to Roche-NimbleGen, Inc. (Madison, WI, USA) for labeling and hybridization onto a human hg18 high-density custom-designed oligonucleotide array (50-mers) containing 2.6 million loci. HpaII amplifiable fragments are defined as genomic sequences contained between two flanking HpaII sites found within 200 to 2,000 bp of each other. All microarray hybridizations were subjected to extensive quality control using the following strategies. First, uniformity of hybridization was evaluated using a modified version of a previously published algorithm [30] adapted for the NimbleGen platform, and any hybridization with strong regional artifacts was discarded and repeated. The raw data can be accessed under GSE49557.

### HELP data processing and analysis

Signal intensities at each HpaII amplifiable fragment were calculated as a robust (25% trimmed) mean of their component probe-level signal intensities. Any fragments found within the level of background MspI signal intensity, measured as 2.5 mean absolute differences (MAD) above the median of random probe signals, were categorized as 'failed'. These 'failed' loci therefore represent the population of fragments that did not amplify by PCR, whatever the biological (for example, genomic deletions and other sequence errors) or experimental cause. On the other hand, 'methylated' loci were so designated when the level of HpaII signal intensity was similarly indistinguishable from background. PCR-amplifying fragments (those not flagged as either 'methylated' or 'failed') were normalized using an intra-array quantile approach wherein HpaII/MspI ratios are aligned across density-dependent sliding windows of fragment size-sorted data. The log_2_ (HpaII/MspI) was used as a representative for methylation and analyzed as a continuous variable. For most loci, each fragment was categorized as either methylated, if the centered log HpaII/MspI ratio was less than zero, or hypomethylated if the log ratio was greater than zero.

Statistical analysis of HELP data was performed using the statistical software R version 2.13.1 [30]. A two-sample *t*-test was used for each gene or locus to summarize methylation differences between the two clinical groups (cases and controls). Genes were ranked on the basis of the magnitude of this test statistic and a set of differentially methylated loci with *P*-value <0.01 and a fold change >0.5 was identified.

### Quantitative DNA methylation analysis by MassArray epityping

Validation of HELP microarray findings was carried out by matrix-assisted laser desorption/ionisation-time of flight (MALDI-TOF) mass spectrometry using EpiTyper by MassArray (Sequenom, San Diego, CA, USA) on bisulfite-converted DNA as previously described [31]. MassArray primers were designed to cover the flanking HpaII sites for a given HpaII-amplifiable fragments (HAF), as well as any other HpaII sites found up to 2,000 bp upstream of the downstream site and up to 2,000 bp downstream of the upstream site, in order to cover all possible alternative sites of digestion. HAF is defined by those fragments where two HpaII sites are located 200–2000 bp apart with at least some unique sequence between them and selected those located at gene promoters and imprinted regions.

### Gene expression analysis using Affymetrix arrays

Transcript levels were analyzed using Affymetrix U133A and 1.0ST arrays. Probes were prepared using an Affymetrix 3′ IVT kit. After hybridization and scanning, raw data files were imported into Genespring GX software (Agilent Technologies). Raw expression levels were normalized using the RMA16 summarization algorithm. Genespring GX software was then used for statistical analysis; the data were above the 20th percentile when filtered by expression. We used a Benjamini-Hochberg multiple testing correction with a *P*-value <0.05. Both heatmap of methylation data and gene expression data were generated using an unsupervised hierarchical clustering method calculated by squared Euclidean distances. Methylation data used in clustering have a *P*-value <0.00015 and a fold change ≥0.5. The raw data can be accessed through accession GSE48944.

### Gene ontology and transcription factor binding sites

The Database for Annotation, Visualization and Integrated Discovery (DAVID) bioinformatics package was used for gene ontology and pathway analysis. In addition, Ingenuity Pathway Analysis (IPA, Redwood City, CA, USA) was used to generate networks.

Sequences of DMRs (n = 4,751) were lifted over from hg18 to hg19 using UCSC Genome Browser Utilities. The regions were then intersected with fetal kidney or human kidney epithelial-specific DHS peaks (data from GEO GSM530655); a total of 364 overlapping regions were used. Motif weight matrices overrepresented in the overlapped sequences were identified using MEME version 4.8.0 [21] on the 364 regions with parameter -oc -nmotifs 10 -minw 8 -maxw 50.

Adult kidney ChIP-seq data were downloaded from the Roadmap database (GEO accessions GSM670025 for adult kidney and GSM621638 for adult kidney input). The overlap was set to be a minimum of 1 bp in length.

### Motif searching

We compared *de novo* motifs to motifs available as part of various databases, including Transfac, version 2011.1, Jaspar Core, and UniPROBE using TOMTOM software [22], version 4.8.1. TOMTOM parameters were set to their default values during motif comparisons. When partitioning the *de novo* motifs, assigning each to a single category, the order of match assignment preference was to Transfac, Jaspar Core, UniPROBE, and then to the novel motif category.

### Cell lines

HKC8 cells were kindly provided by Lorainne Racusen (Johns Hopkins University) and were cultured in DMEM/F12 medium supplemented with 2.5% fetal bovine serum, antibiotics and insulin, transferrin and selenium. Cells were incubated with 0.5 μM decitabine on days 2, 4, 6, and 8 and harvested on day 9. RNA was isolated using a Qiagen RNeasy kit labeled using an Ovation transcript labeling kit and hybridized onto Affymetrix Human ST1.0 arrays.

### Chromatin immunoprecipitation sequencing

HKC8 cells were harvested and crosslinked with 1% formaldehyde when they reached 80% confluency on culture plates. Chromatin was sheared using a Bioruptor and immunoprecipitated using H3K4me1 (Abcam ab8895, Cambridge, MA, USA), H3K4me2 (Abcam ab11946), H3K4me3 (Abcam ab8580), H3K36me3 (Abcam ab9050), H3K27ac (Abcam ab4729) and H3K27me3 (Millipore 07–499, Billerica, MA, USA) marks. ChIP was performed as described in the manual of MAGnify™ Chromatin Immunoprecipitation System (Invitrogen, Grand Island, NY, USA). Quantitative real-time PCR was performed to ensure the quality of the ChIP product. The ChIP product was assessed for size, purity, and quantity using an Agilent 2100 Bioanalyzer (Agilent Technologies). Library preparation and sequencing were performed at the Einstein Epigenome Center. Sequence reads (100 bp) were generated from llumina HiSeq 2000 [32]. Reads were aligned to the reference genomes (NCBI build 37, hg19) using Bowtie (v 0.12.7). Repetitively mapped and duplicate reads were excluded. The data can be accessed using accession GSE49637.

### ChIP-seq data analysis

We used the MACS version 1.4.1 (model-based analysis of ChIP-Seq) peak-finding algorithm to identify regions of ChIP-Seq enrichment over background [33]. A false discovery rate threshold of enrichment of 0.01 was used for all data sets. The resulting genomic coordinates in bed format were further used in ChromHMM v1.06 for chromatin annotation. The following parameters were used: -Xmx1600M -jar ChromHMM.jar BinarizeBed hg19 -Xmx2000M -jar ChromHMM.jar LearnModel 10 hg19.

### DNase I hypersensitive site analysis

Human kidney DHS sequencing data (GEO GSM530655) was analyzed with MACS (v.1.4.1). The resulting peaks were overlapped with the differentially methylated regions. The control random genomic loci were generated using Regulatory Sequence Analysis Tools. Based on the data property of differentially methylated regions, we used the same number of fragments (4,751) and the same average fragment size (443 bp) as parameters for the random loci.

### Illumina infinium 450K BeadChip arrays

Genomic DNA (200 ng) was purified using the DNeasy Blood and Tissue Kit (Qiagen) following the manufacturer’s protocol. Purified DNA quality and concentration were assessed with a NanoDrop ND-1000 (Thermo Scientific, Waltham, MA, USA) and by Quant-iT™ PicoGreen^®^ dsDNA Assay Kit (Life Technologies) prior to bisulfite conversion. Purified genomic DNA was bisulfite converted using the EZ DNA Methylation Kit (Zymo Research, Orange, CA, USA) following the manufacturer’s protocol. Bisulfite DNA quality and concentration were assessed, following the Illumina 450K array protocol, bisulfite converted sample was whole-genome amplified, enzymatically digested, and hybridized to the array, and then single nucleotide extension was performed.

Chips were scanned using an Illumina HiScan on a two-color channel to detect Cy3-labeled probes on the green channel and Cy5-labeled probes on the red channel. Illumina GenomeStudio Software 2011.1 Methylation Module 1.8 was used to read the array output and conduct background normalization. The level of DNAm for 428,216 probes in our sample dataset was intersected with the expanded annotation for further analyses. All samples were run together to eliminate the batch effect according to the pipelines established by Illumina Genome Studio. The full dataset can be accessed in GEO under GSE50874.

## Abbreviations

bp: base pair; CKD: chronic kidney disease; ChIP: chromatin immunoprecipitation; DHS: DNase I hypersensitivity; DKD: diabetic CKD; DMR: differentially methylated region; GEO: Gene Expression Omnibus; HELP: HpaII fragment enrichment by ligation-mediated PCR; TGFβ: transforming growth factor beta; UTR: untranslated region.

## Competing interests

The authors declare that they have no competing interests.

## Authors’ contributions

YAK performed the ChIP-seq experiments and analyzed the data. DM, MS, ASP, HS, and SYH collected the samples and performed the human kidney experiments. MCI and HMK helped with the *in vivo* studies. JP helped with tissue collection and performed the histological analysis. TH helped with the clinical studies. AV and DZ helped with the analysis. JMG and KS oversaw the studies and wrote the manuscript. All authors read and approved the final manuscript.

## Supplementary Material

Additional file 1: Table S1 Demographic, clinical and histological characteristics of the samples. Click here for file

Additional file 2: Figure S1 Principal component analysis of the transcript levels in the original dataset show no significant differences based on diabetes status of the samples. Dark red circles indicate CKD gene expression data points, light red circles indicate DKD data points, light blue diabetic control data points and dark blue control data points. Click here for file

Additional file 3: Table S2 List of differentially methylated regions in CKD. List of differentially methylated loci, *P*-values, genomic location (hg18), and their methylation levels in individual samples, with the nearest annotated transcript to each DMR listed. Click here for file

Additional file 4: Figure S2 MassArray-based confirmation of cytosine methylation levels. Absolute methylation values are plotted on the y-axis while relative methylation values from the HELP dataset are shown on the x-axis. Each plot represents methylation values from one human kidney tissue (HK). We ran each sample with nine different primer sets that represent low, intermediate and highly methylated regions. Click here for file

Additional file 5: Table S3 External validation of HELP DMRs using the Illumina Infinium 450K platform of 87 DKD samples. The CKD (HELP) DMRs were assigned to the nearest RefSeq genes and the methylation differences for these RefSeq genes were extracted from the Infinium 450K arrays. The probes showing differential methylation are listed here. Multiple probes presented for the HELP DMRs were also differentially methylated in the Infinium 450K arrays. The number of gene-based, unique overlapping DMRs was 1,061. Click here for file

Additional file 6: Figure S3 MassArray-based confirmation and external validation of the differentially methylated loci. **(A,D)** Average HpaII/MspI methylation ratio of DMRs on the HELP array in control (blue) and CKD kidneys (red). The original data can be found in Additional file 3. **(B,E)** MassArray Epityper-based absolute methylation level of the locus for control (blue) and CKD kidneys (red). Note that one HELP probe represents multiple CpG sites. **(C,F)** Methylation difference between DKD and control for this region in the external validation dataset. This dataset was generated using the Illumina Infinium 450K arrays from 66 control and 21 DKD microdissected kidney samples. The original data can be found in Additional file 7). (A-C) Changes in the Dermatopontin gene (DPT); (D-F) changes in the Down syndrome cell adhesion molecule (DSCAM) locus. Click here for file

Additional file 7: Figure S4 RefSeq annotation of the DMRs. The number of probes on the Roche-NimbleGen customized array, DMRs, hypo- or hypermethylated DMRs in each Refseq-based annotation groups. Relative enrichment ratio of the DMR compared with the representation of the different elements on the methylation microarray. Click here for file

Additional file 8: Table S4 Correlations of DMR and transcript levels. DMRs from the HELP assay and its corresponding transcripts (Affymetrix arrays) are listed. There were multiple DMRs for some of the transcripts and they are all listed. Chromosomal location, methylation level, gene expression differences, and *P*-values are included in the table. Click here for file

Additional file 9: Table S5 CKD DMRs observed in decitabine-treated cells. CKD DMR loci showing statistically significant differences in HKC8 cells treated with 0.5 μM decitabine. The CKD DMRs were analyzed using HELP assays, while the decitabine-treated cells were analyzed using Infinium arrays. Click here for file
