# Supplementary material for: Cytosine methylation changes in enhancer regions of core pro-fibrotic genes characterize kidney fibrosis development
Source: Genome Biol. 2013 Oct 7;14(10):R108. doi: 10.1186/gb-2013-14-10-r108 (PMC4053753; doi:10.1186/gb-2013-14-10-r108)
Supplement: Additional file 2: Figure S1 — Principal component analysis of the transcript levels in the original dataset show no significant differences based on diabetes status of the samples. Dark red circles indicate CKD gene expression data points, light red circles indicate DKD data points, light blue diabetic control data points and dark blue control data points. [file gb-2013-14-10-r108-S2.pdf]

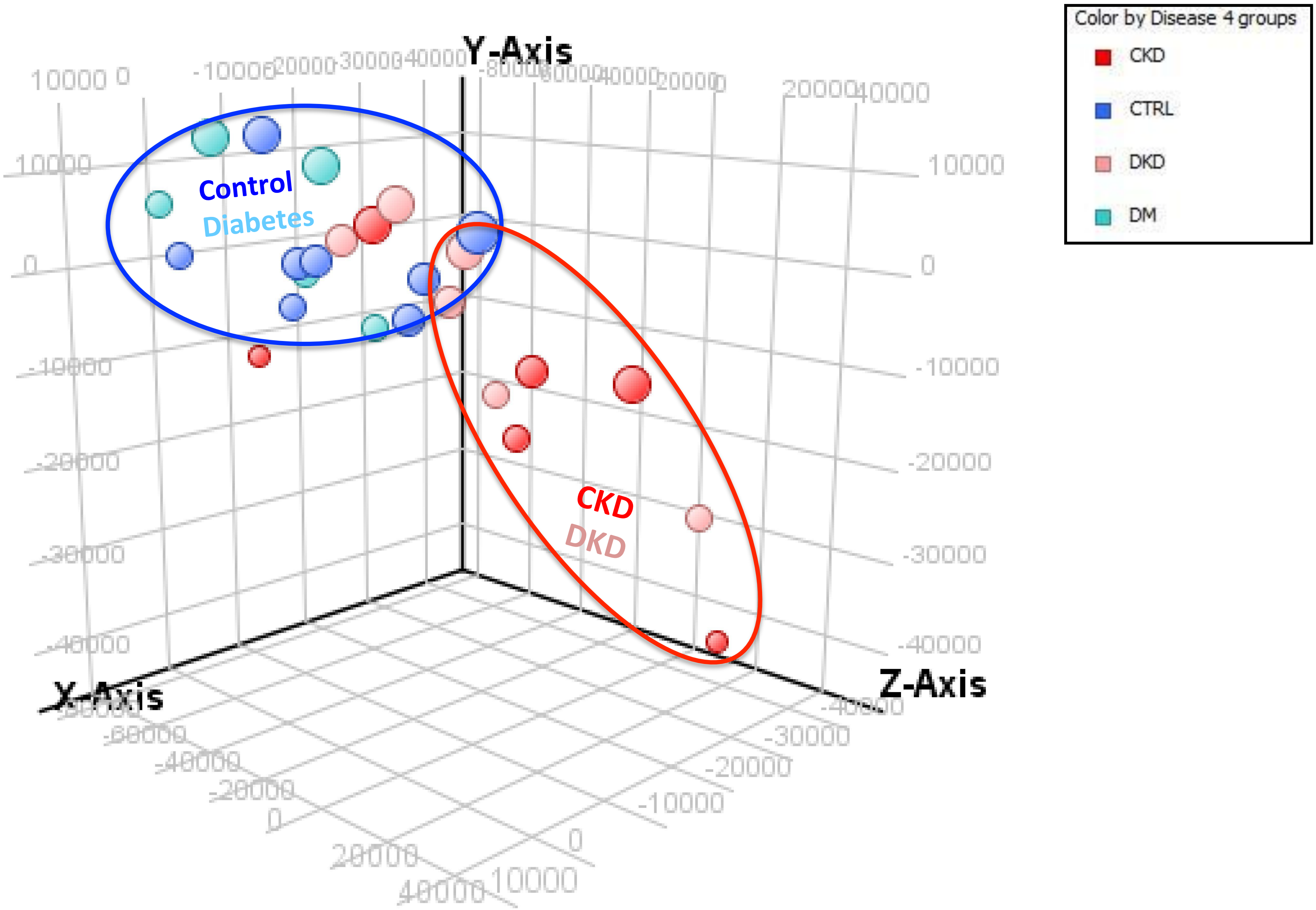

Supplemental Figure1

Supplemental Figure 2: Technical Validation

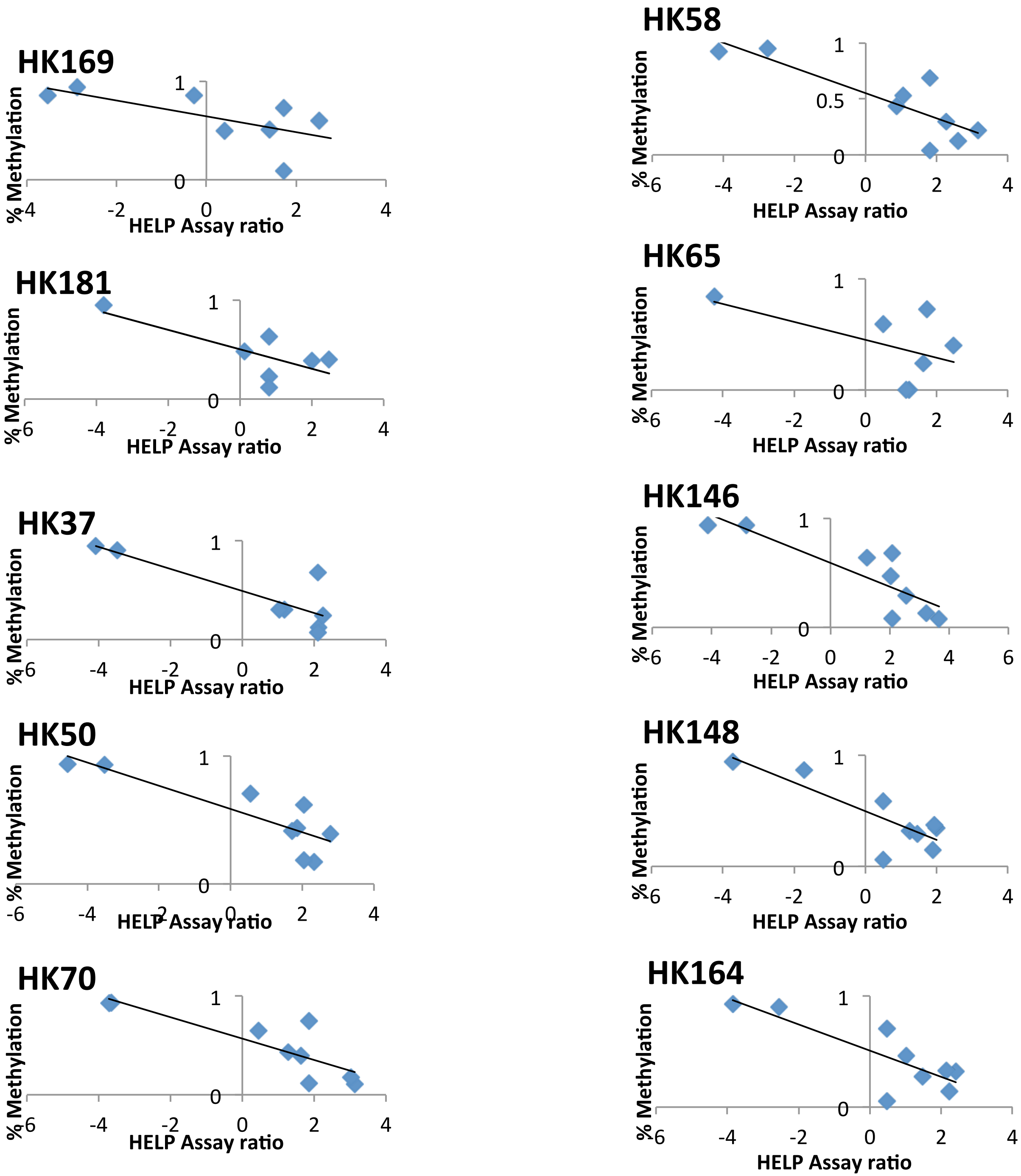

Supplemental Figure 3

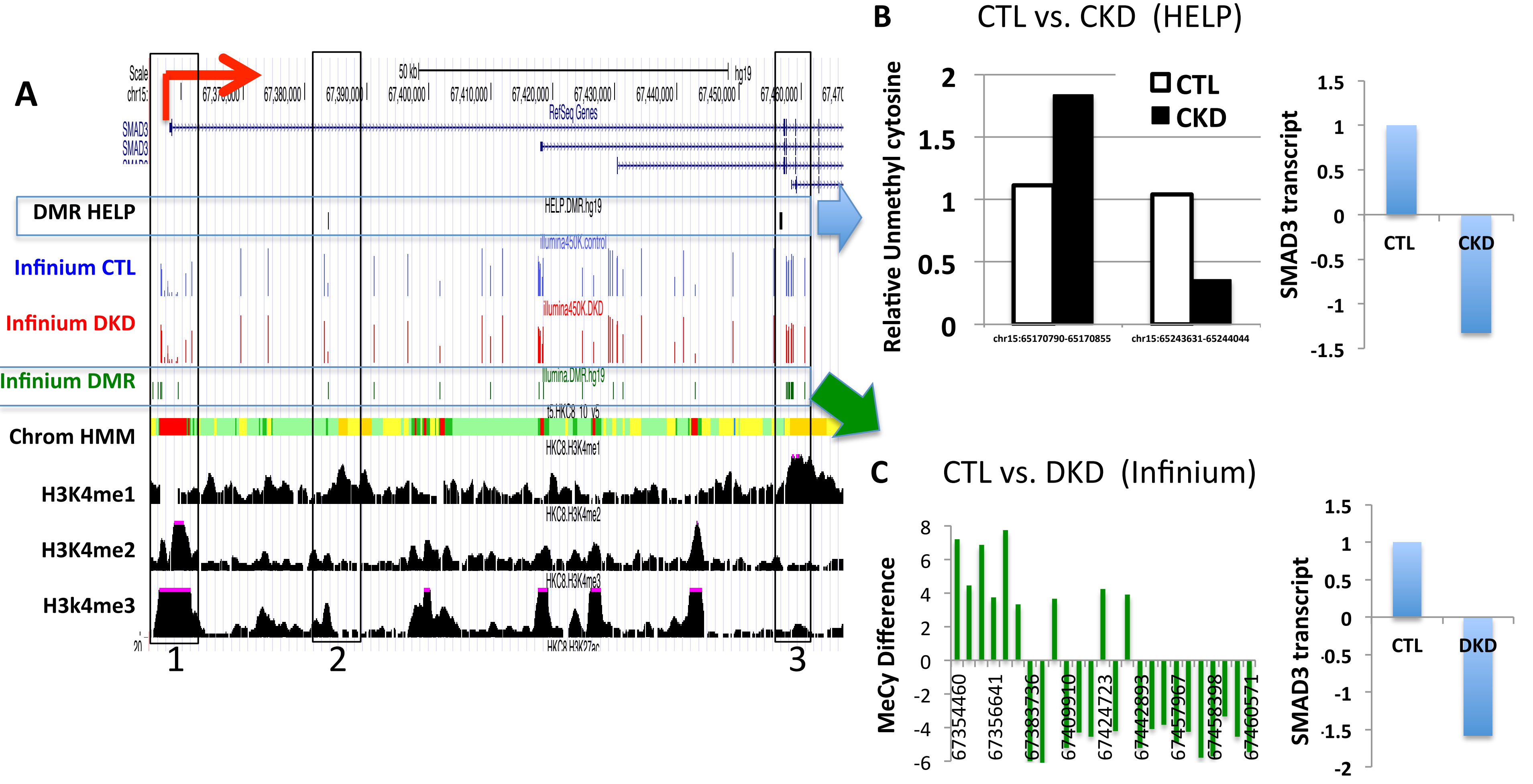

Supplemental Figure 3

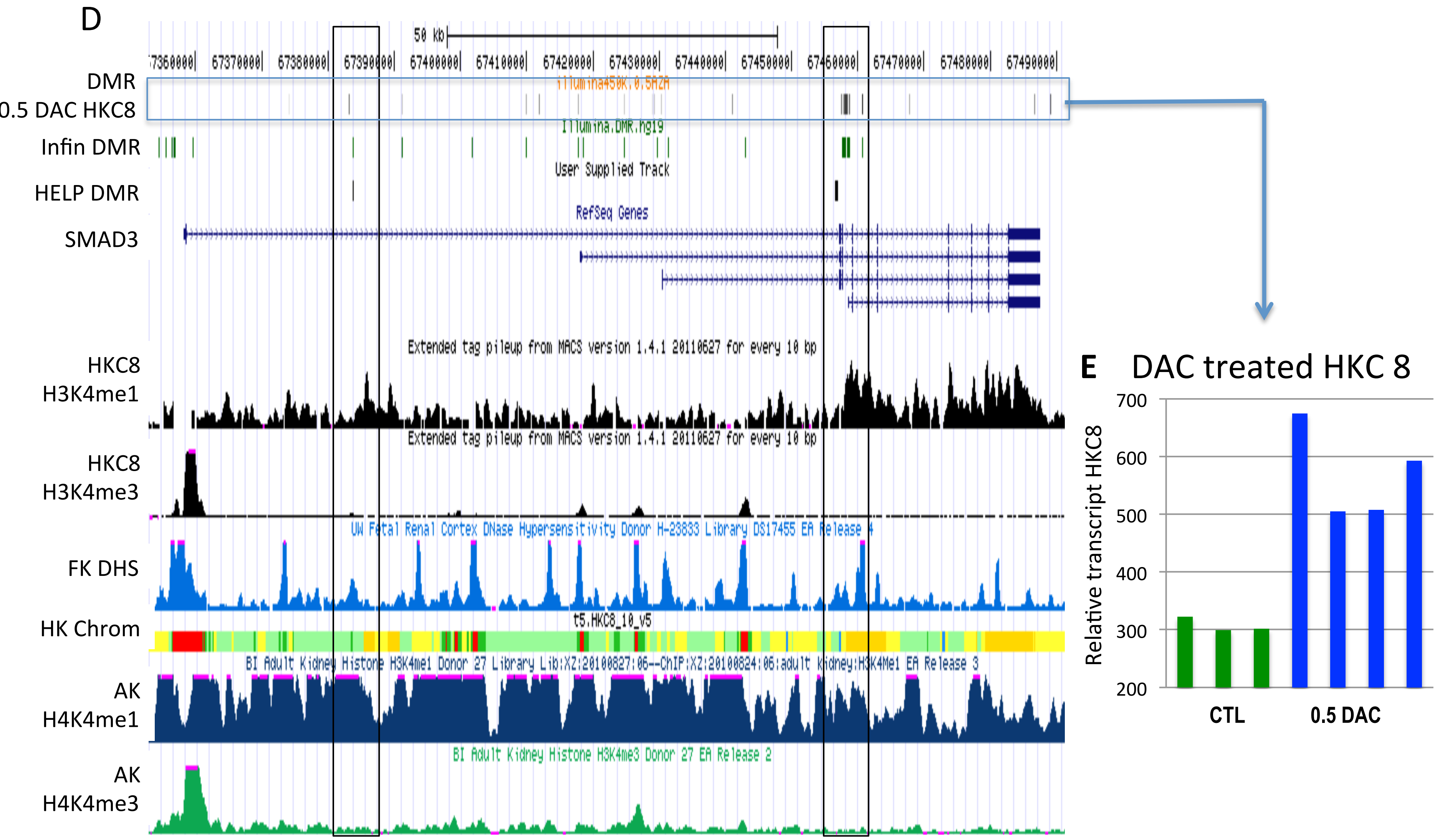

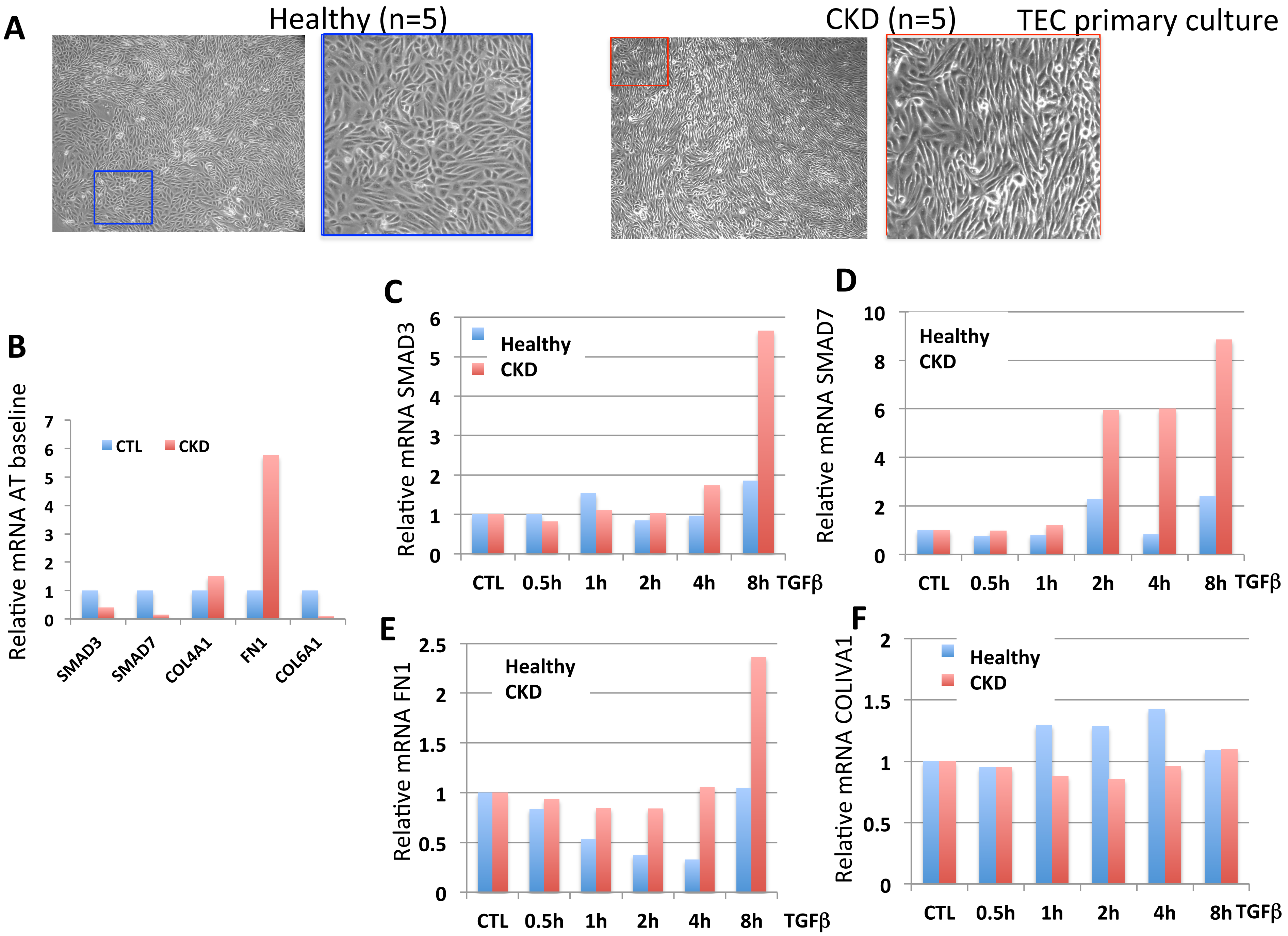

Supplemental Figure 4

Supplemental Figure 4

G

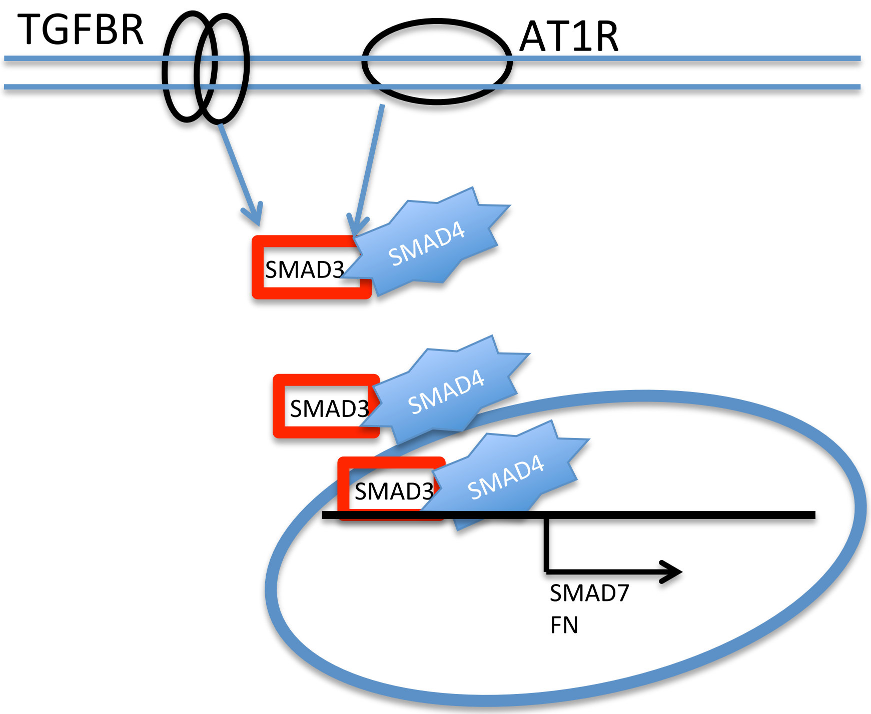

H

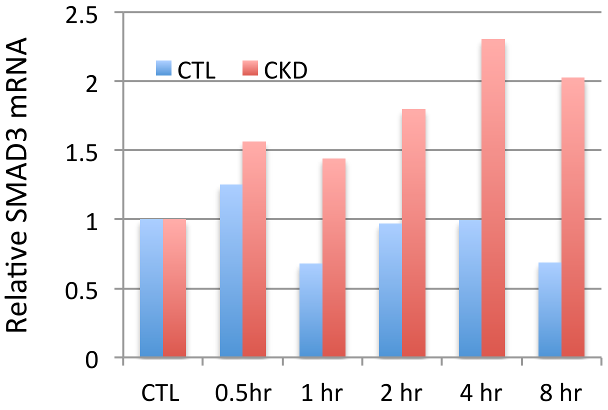

I

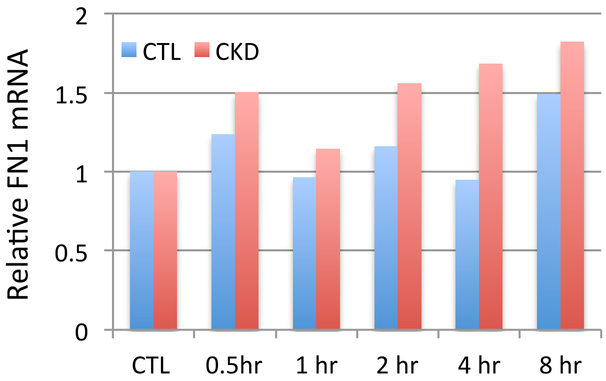

J

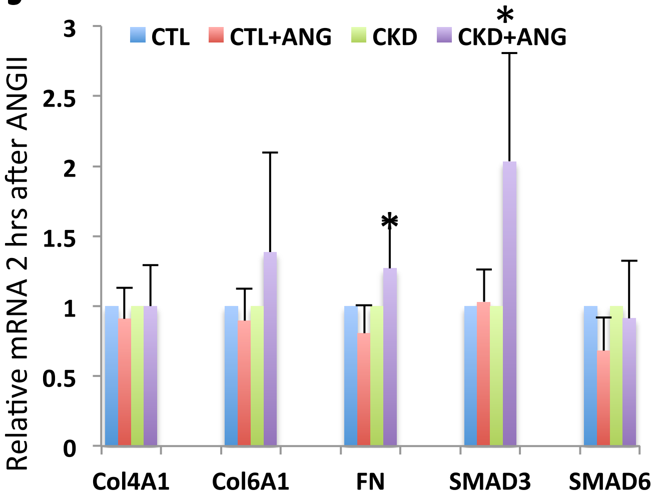

HEALTHY/NORMAL

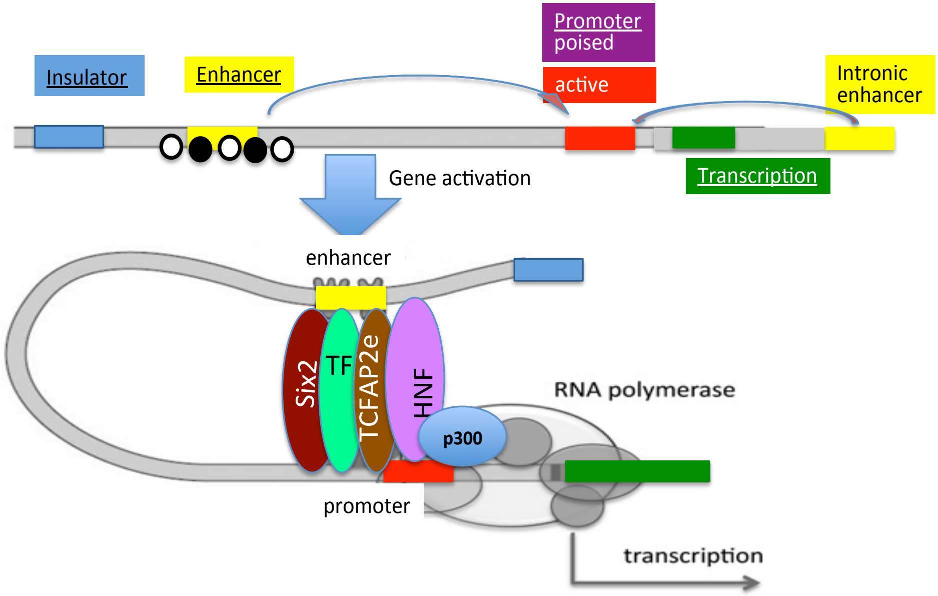

Correct Cytosine methylation
Correct signal strength

CKD

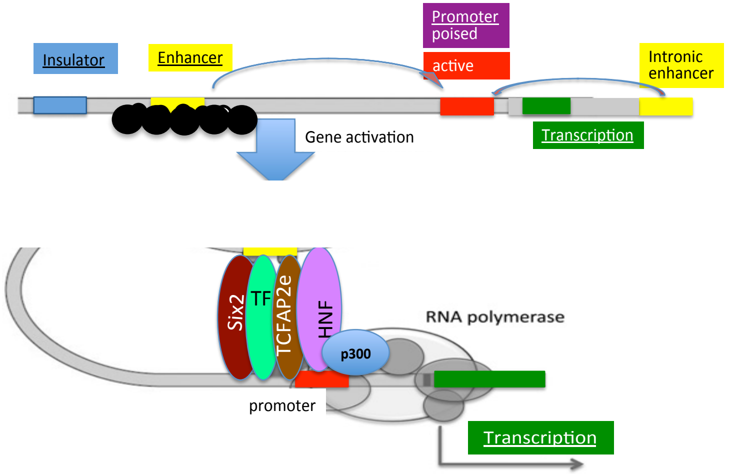

Increased cytosine methylation of enhancer decreased signal strength

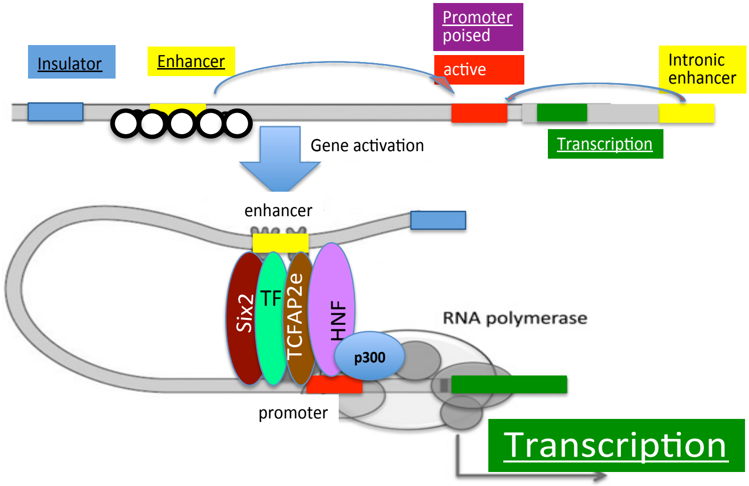

Loss of cytosine methylation of enhancer increased signal strength

Variable/incorrect signal strength

Supplemental Figure 5
