# Supplementary figures and images for: Cytosine methylation changes in enhancer regions of core pro-fibrotic genes characterize kidney fibrosis development
Source: Genome Biol. 2013 Oct 7;14(10):R108. doi: 10.1186/gb-2013-14-10-r108 (PMC4053753; doi:10.1186/gb-2013-14-10-r108)

Supplemental Figure 2: Technical Validation

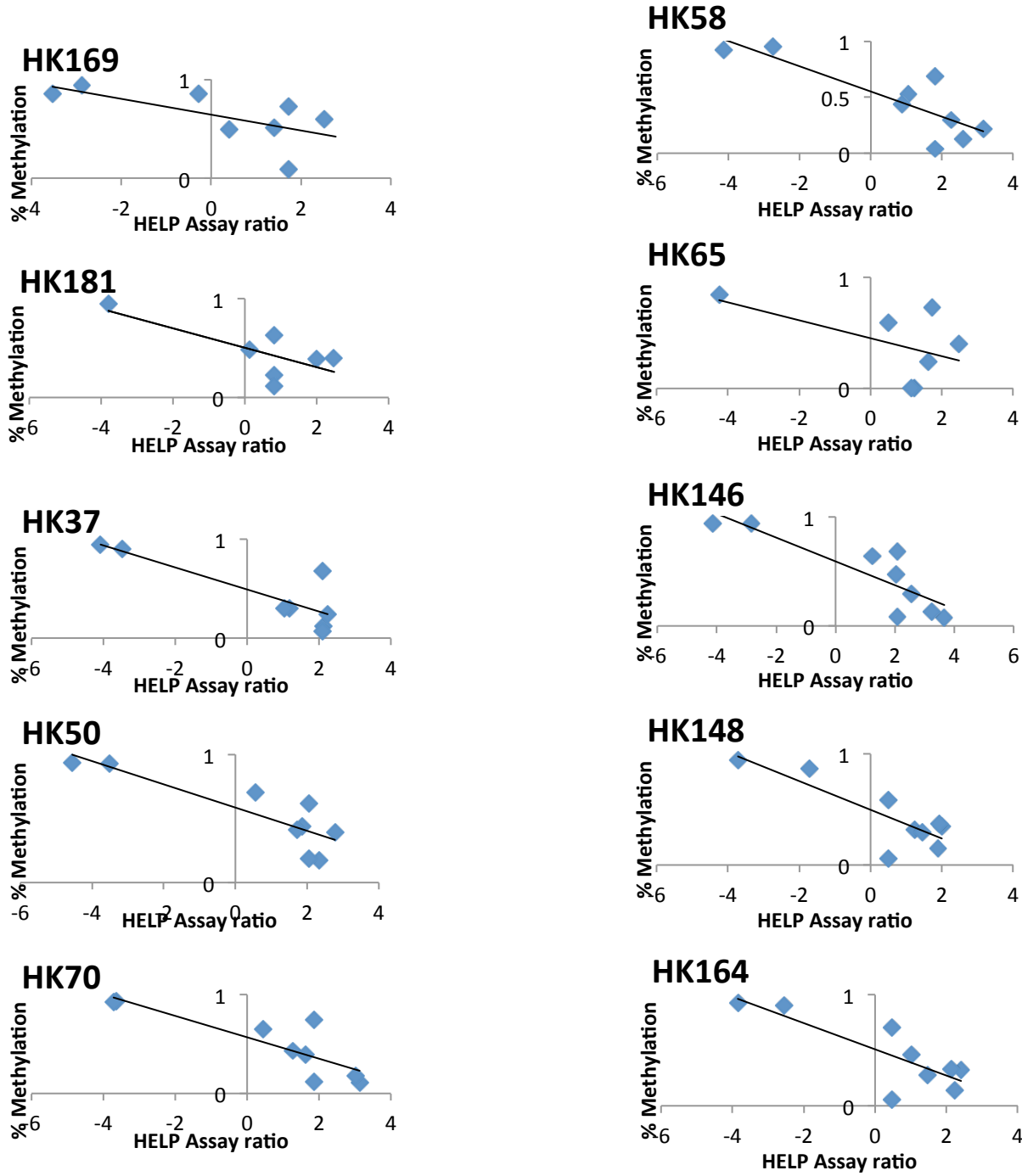

Supplement: Additional file 4: Figure S2 — MassArray-based confirmation of cytosine methylation levels. Absolute methylation values are plotted on the y-axis while relative methylation values from the HELP dataset are shown on the x-axis. Each plot represents methylation values from one human kidney tissue (HK). We ran each sample with nine different primer sets that represent low, intermediate and highly methylated regions. [file gb-2013-14-10-r108-S4.pdf]
