# Supplementary material for: Cytosine methylation changes in enhancer regions of core pro-fibrotic genes characterize kidney fibrosis development
Source: Genome Biol. 2013 Oct 7;14(10):R108. doi: 10.1186/gb-2013-14-10-r108 (PMC4053753; doi:10.1186/gb-2013-14-10-r108)
Supplement: Additional file 6: Figure S3 — MassArray-based confirmation and external validation of the differentially methylated loci. (A,D) Average HpaII/MspI methylation ratio of DMRs on the HELP array in control (blue) and CKD kidneys (red). The original data can be found in Additional file 3. (B,E) MassArray Epityper-based absolute methylation level of the locus for control (blue) and CKD kidneys (red). Note that one HELP probe represents multiple CpG sites. (C,F) Methylation difference between DKD and control for this region in the external validation dataset. This dataset was generated using the Illumina Infinium 450K arrays from 66 control and 21 DKD microdissected kidney samples. The original data can be found in Additional file 7). (A-C) Changes in the Dermatopontin gene (DPT); (D-F) changes in the Down syndrome cell adhesion molecule (DSCAM) locus. [file gb-2013-14-10-r108-S6.pptx]

## Slide 1
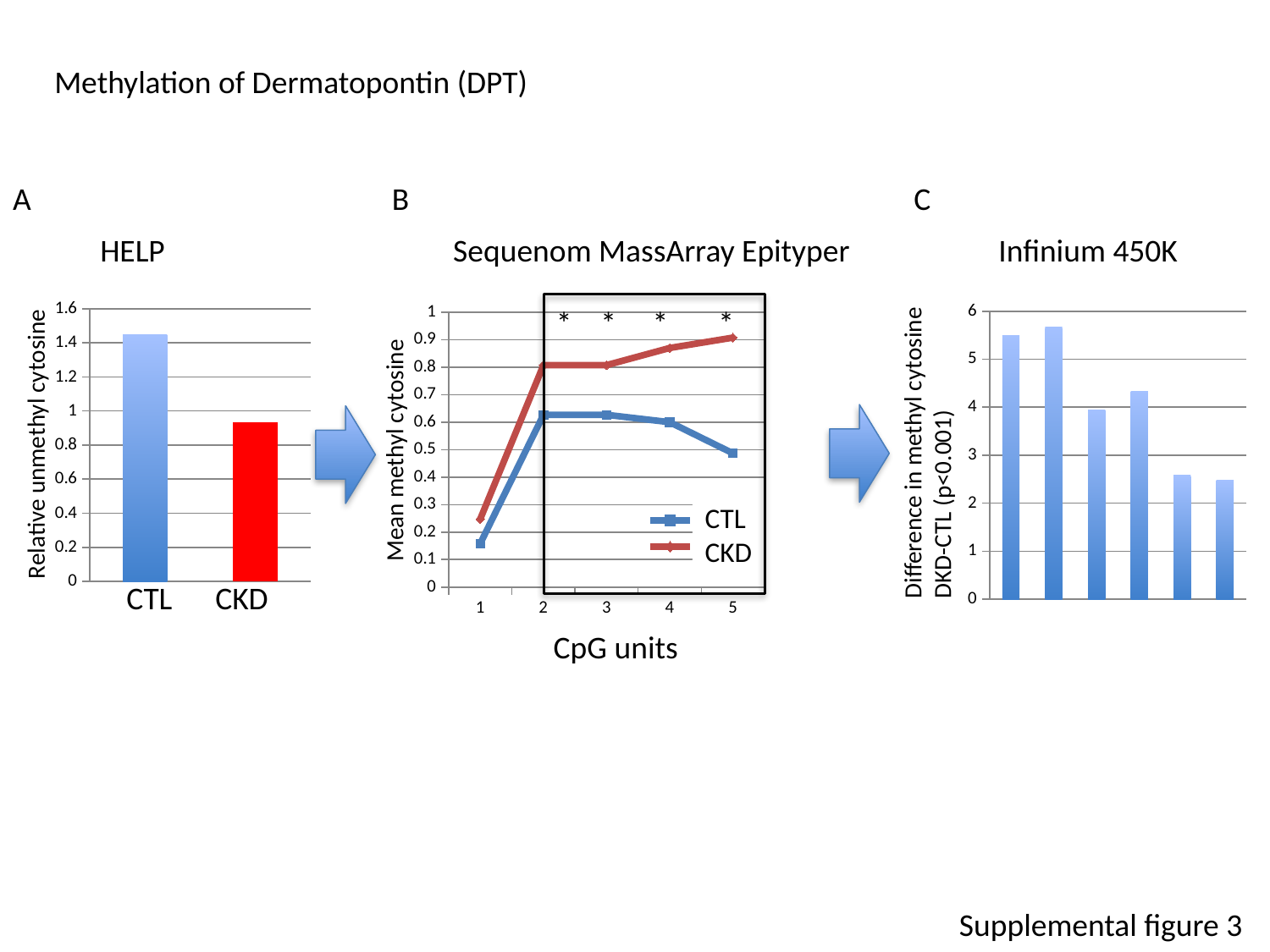

Methylation of Dermatopontin (DPT)
A
B
C
HELP
Sequenom MassArray Epityper
Infinium 450K
### Chart
| Category | | |
|---|---|---|
### Chart
| Category | |
|---|---|
### Chart
| Category | |
|---|---|* * * *
Difference in methyl cytosine
DKD-CTL (p<0.001)
Relative unmethyl cytosine
Mean methyl cytosine
CTL
CKD
CTL CKD
CpG units
Supplemental figure 3

## Slide 2
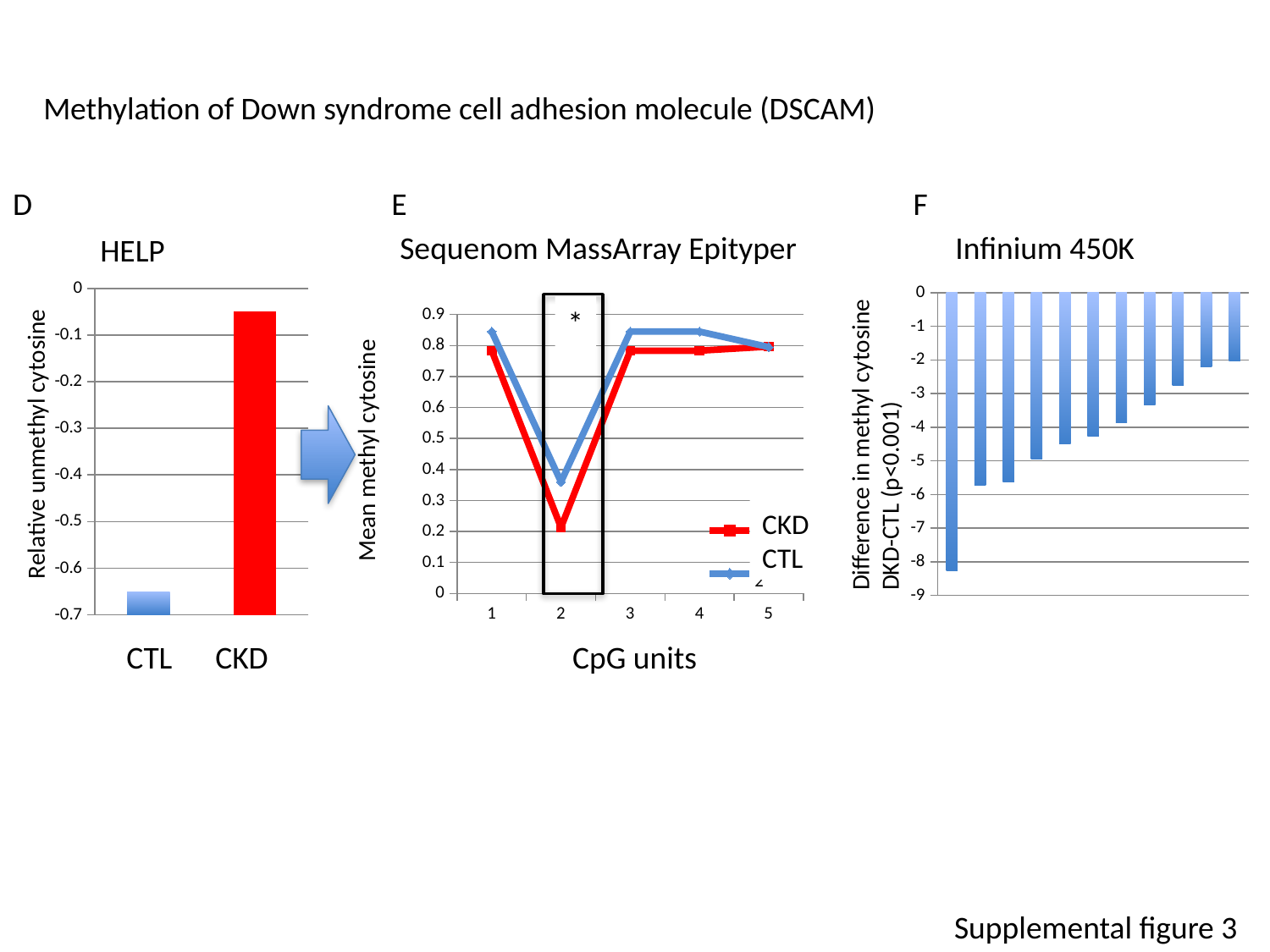

Methylation of Down syndrome cell adhesion molecule (DSCAM)
D
E
F
Sequenom MassArray Epityper
Infinium 450K
HELP
### Chart
| Category | |
|---|---|
### Chart
| Category | |
|---|---|
### Chart
| Category | | |
|---|---|---|
*
Difference in methyl cytosine
DKD-CTL (p<0.001)
Relative unmethyl cytosine
Mean methyl cytosine
CKD
CTL
CpG units
CTL CKD
Supplemental figure 3
