# Supplementary material for: Cytosine methylation changes in enhancer regions of core pro-fibrotic genes characterize kidney fibrosis development
Source: Genome Biol. 2013 Oct 7;14(10):R108. doi: 10.1186/gb-2013-14-10-r108 (PMC4053753; doi:10.1186/gb-2013-14-10-r108)
Supplement: Additional file 7: Figure S4 — RefSeq annotation of the DMRs. The number of probes on the Roche-NimbleGen customized array, DMRs, hypo- or hypermethylated DMRs in each Refseq-based annotation groups. Relative enrichment ratio of the DMR compared with the representation of the different elements on the methylation microarray. [file gb-2013-14-10-r108-S7.pptx]

## Slide 1
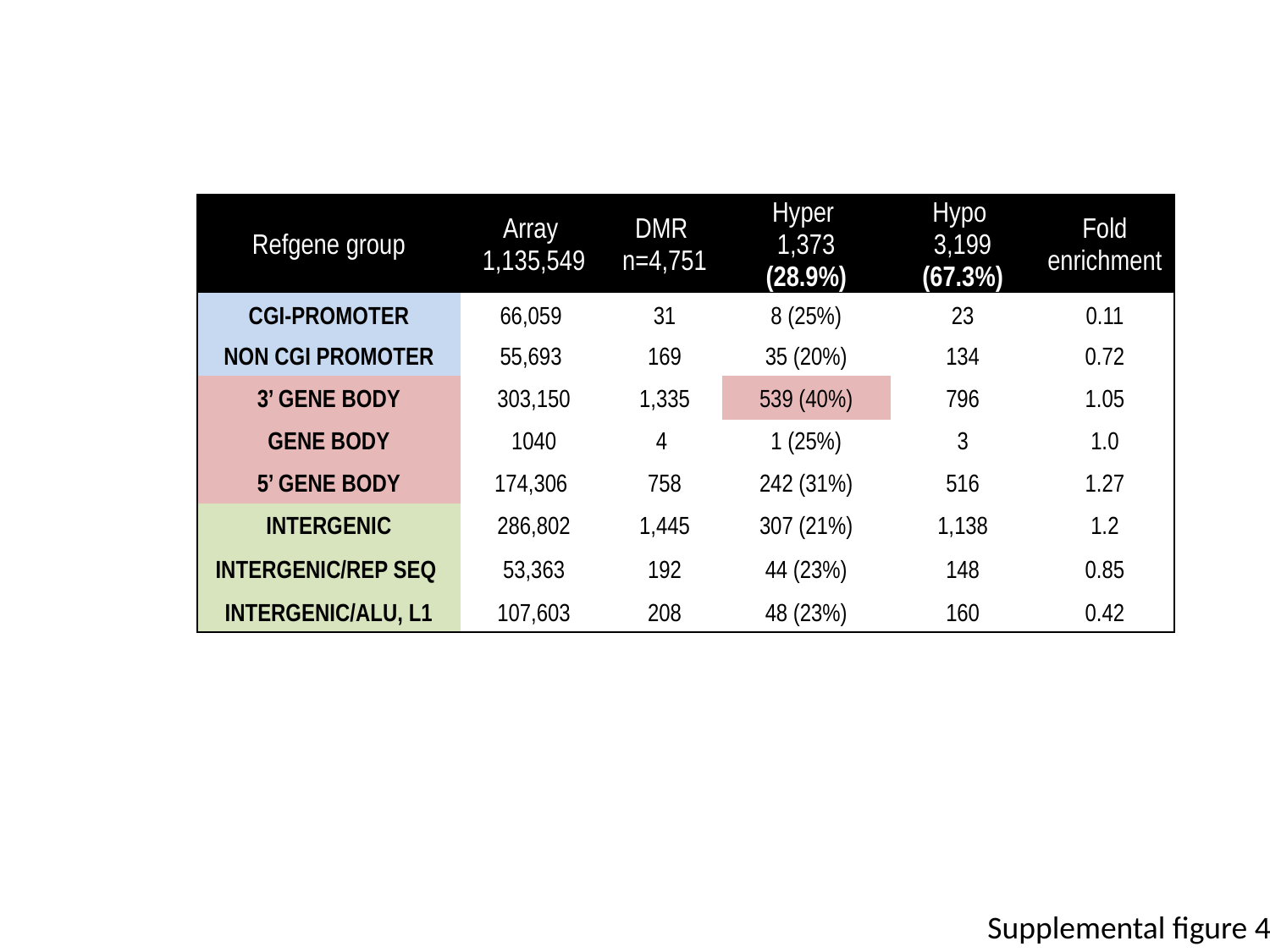

| Refgene group | Array 1,135,549 | DMR n=4,751 | Hyper 1,373 (28.9%) | Hypo 3,199 (67.3%) | Fold enrichment |
| --- | --- | --- | --- | --- | --- |
| CGI-Promoter | 66,059 | 31 | 8 (25%) | 23 | 0.11 |
| Non CGI promoter | 55,693 | 169 | 35 (20%) | 134 | 0.72 |
| 3’ gene body | 303,150 | 1,335 | 539 (40%) | 796 | 1.05 |
| Gene body | 1040 | 4 | 1 (25%) | 3 | 1.0 |
| 5’ gene body | 174,306 | 758 | 242 (31%) | 516 | 1.27 |
| Intergenic | 286,802 | 1,445 | 307 (21%) | 1,138 | 1.2 |
| Intergenic/rep seq | 53,363 | 192 | 44 (23%) | 148 | 0.85 |
| Intergenic/Alu, L1 | 107,603 | 208 | 48 (23%) | 160 | 0.42 |
Supplemental figure 4
